# BRCA1 Is a Histone-H2A-Specific Ubiquitin Ligase

**DOI:** 10.1016/j.celrep.2014.07.025

**Published:** 2014-08-14

**Authors:** Reinhard Kalb, Donna L. Mallery, Conor Larkin, Jeffrey T.J. Huang, Kevin Hiom

**Affiliations:** 1 Division of Protein Nucleic Acid Chemistry, MRC Laboratory of Molecular Biology, Francis Crick Avenue, Cambridge Biomedical Campus, Cambridge CB2 0QH, UK; 2 Department of Chromatin Research, MPI of Biochemistry, Am Klopferspitz 18, 82152 Martinsried, Germany; 3 Division of Cancer Research, Medical Research Institute, Ninewells Hospital & Medical School, Dundee DD1 9SY, Scotland; 4 Biomarker and Drug Analysis Core Facility, Medical Research Institute, Ninewells Hospital & Medical School, Dundee DD1 9SY, Scotland

## Abstract

The RING domain proteins BRCA1 and BARD1 comprise a heterodimeric ubiquitin (E3) ligase that is required for the accumulation of ubiquitin conjugates at sites of DNA damage and for silencing at DNA satellite repeat regions. Despite its links to chromatin, the substrate and underlying function of the BRCA1/BARD1 ubiquitin ligase remain unclear. Here, we show that BRCA1/BARD1 specifically ubiquitylates histone H2A in its C-terminal tail on lysines 127 and 129 in vitro and in vivo. The specificity for K127-129 is acquired only when H2A is within a nucleosomal context. Moreover, site-specific targeting of the BRCA1/BARD1 RING domains to chromatin is sufficient for H2Aub foci formation in vivo. Our data establish BRCA1/BARD1 as a histone-H2A-specific E3 ligase, helping to explain its localization and activities on chromatin in cells.

## Highlights

• BRCA1/BARD1 is a histone-H2A-specific ubiquitin ligase
• BRCA1/BARD1 ubiquitylates histone H2A on lysines 127 and 129
• Specificity for H2A is provided within in a nucleosomal context
• Heterodimeric RING domains of BRCA1/BARD1 are sufficient for E3 function in vivo

## Graphical Abstract

**Figure:**
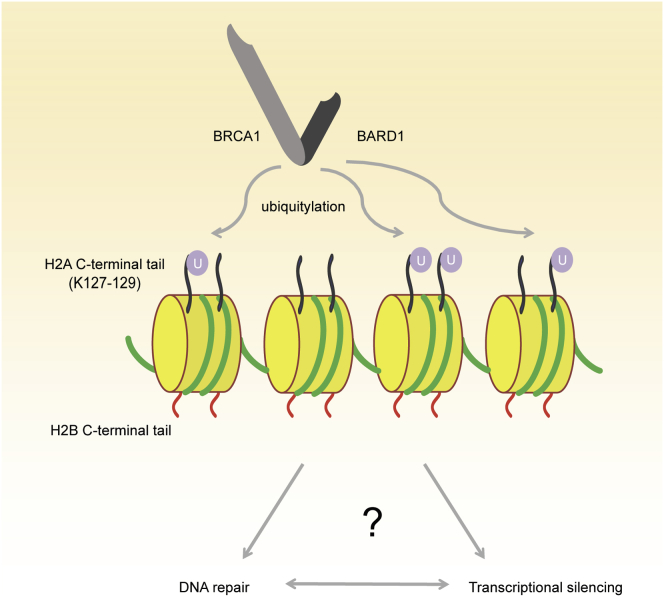

## Teaser

The ubiquitin ligase (E3) activity of BRCA1 is its only known biochemical activity in vitro. Kalb et al. report that the heterodimeric complex formed by BRCA1/BARD1 ubiquitylates histone H2A in nucleosomes specifically at its C-terminal tail. Moreover, in vitro and in vivo assays identify H2A lysines127 and 129 as the target lysines. These results help to explain the localization and activity of BRCA1/BARD1 on chromatin in cells.

## Introduction

Breast cancer-associated protein 1 (BRCA1) is a key mediator in the DNA damage response, which is linked to a wide range of functions that serve to maintain genomic stability. Cellular BRCA1 forms a heterodimer with BRCA1-associated RING domain 1 (BARD1) (Wu et al., 1996), which promotes the repair of double-stranded DNA breaks through homologous recombination (Moynahan and Jasin, 2010) and contributes to the DNA-damage-induced G2/M checkpoint (Xu et al., 2001). Loss of BRCA1 function in cells results in hypersensitivity to DNA damage and accumulation of chromosomal aberrations associated with the development of cancer (Venkitaraman, 2001).

In vitro studies have shown that the RING domains of BRCA1/BARD1 possess a ubiquitin ligase (E3) function (Ruffner et al., 2001), but a bona fide substrate for this activity is still lacking. During replication and after treatment with agents that damage DNA, BRCA1 and BARD1 colocalize in discreet nuclear foci with ubiquitin conjugates (Morris and Solomon, 2004). However, it is not known whether these conjugates are a product of BRCA1/BARD1 E3 activity or arise through the function of several other E3 proteins that also colocalize at sites of DNA double-strand breaks as part of a ubiquitin-mediated DNA-damage-signaling pathway (Doil et al., 2009; Mattiroli et al., 2012; Stewart et al., 2009).

Recently, Zhu et al. (2011) reported that defects in BRCA1 E3 function are linked with a derepression of satellite DNA that is accompanied by decompaction of chromatin and reduced levels of ubiquitylated histone H2A (H2Aub). Moreover, these phenotypes can be reversed by exogenous expression of histone H2A protein fused to ubiquitin. However, a direct role for BRCA1/BARD1-dependent ubiquitylation of histones was not established.

We investigated the interaction of BRCA1/BARD1 with chromatin and found that it involves a highly specific histone H2A-ubiquitin ligase that modifies previously uncharacterized lysines in the C-terminal tail of H2A. We discuss these observations in light of the known biological functions of BRCA1.

## Results

### BRCA1/BARD1 Ubiquitylates H2A in Nucleosomes

Although BRCA1 binds to DNA in a sequence-independent manner in vitro (Paull et al., 2001), in cells, it is most commonly found at DNA breaks associated with chromatin. Accordingly, we found that purified recombinant BRCA1/BARD1 bound nucleosome substrates in an electrophoretic mobility shift assay (EMSA) (Figures S1A–S1F). The binding was dynamic, as complexes were competed away by addition of unlabeled DNA (Figure S1D). This binding, along with the known structural similarities between the RING domains of BRCA1/BARD1 and those of the histone-H2A-specific ubiquitin ligase complex Polycomb repressive complex 1 (PRC1) (Buchwald et al., 2006; Figure S1G), prompted us to investigate whether BRCA1/BARD1 ubiquitylates nucleosomal histone proteins.

**Figure 1:**
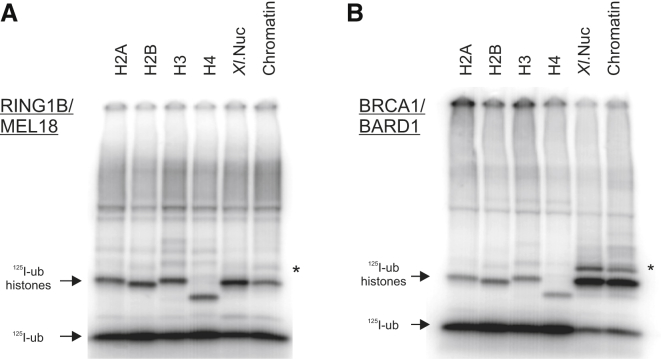
BRCA1/BARD1 Specifically Ubiquitylates Histone H2A in Nucleosomes (A and B) Ubiquitylation of recombinant *Xenopus laevis* histones (H2A, H2B, H3, and H4), reconstituted nucleosomes, and chromatin isolated from HeLa cells by RING1B/MEL18 (A) or BRCA1/BARD1 (B) ubiquitin ligases. ^125^I-labeled ubiquitin is covalently linked to its substrate and detected after SDS-PAGE. Specificity for a single histone occurs only within a nucleosomal context. Small amounts of diubiquitylated histone were observed as indicated (^∗^). See also Figure S1.

We examined BRCA1/BARD1 E3 activity on individual histone proteins and reconstituted nucleosome substrates in vitro and compared it with the activity of the RING1B/MEL-18 subunits of PRC1 (Figures 1A and 1B). As reported previously, RING1B/MEL18 monoubiquitylated individual histone proteins with similar efficiency in vitro, but ubiquitylated nucleosome substrates specifically on K118-119 of H2A (Figure 1A). This reflects the known cellular specificity of PRC1 for K118-119 of H2A (Elderkin et al., 2007; Wang et al., 2004). BRCA1/BARD1 acted similarly, ubiquitylating individual H2A, H2B, H3, and H4 proteins in vitro without any apparent preference (Mallery et al., 2002; Figure 1B). However, when BRCA1/BARD1 was incubated with recombinant nucleosomes or native chromatin, only H2A was ubiquitylated (Figure 1B). We concluded that BRCA1/BARD1 E3 activity resembles that of PRC1 in that it acquires specificity for histone H2A as a substrate when the latter is incorporated into nucleosomes.

### BRCA1/BARD1 RING Domains Ubiquitylate H2A In Vivo

**Figure 2:**
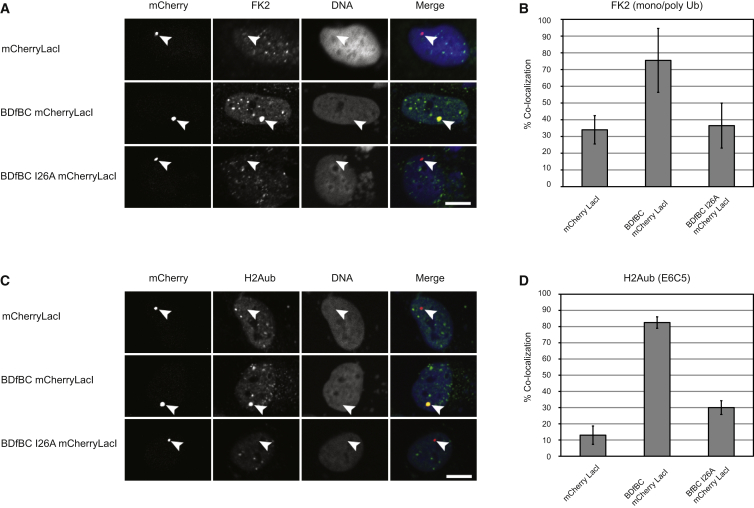
Ubiquitylation of Histone H2A by BRCA1/BARD1 E3 Activity In Vivo (A and C) Site-specific recruitment of the BRCA1/BARD1 E3 to a single genomic location in HEK293 2-6-3 cells results in ubiquitylation of histone H2A. U2OS 2-6-3 cells containing 200 tandem copies of a 256 LacO sequence integrated at a specific site were transiently transfected with plasmids expressing mCherryLacI, BDfBC-mCherryLacI, or mutant BDfBC-I26A-mCherryLacI. (A and B) Cells were stained with antibodies against H2Aub (E6C5; A) and scored for colocalization with mCherry (B). (C and D) Cells were stained with antibodies against ubiquitin (FK2; C) and scored for colocalization with mCherry (D). Representative images are shown. Values represent the mean from two independent experiments (n = 100). Error bars represent 1 SD. Scale bar represents 10 μm. See also Figure S2.

The recruitment of BRCA1/BARD1 to sites of DNA damage is dependent on the activity of several other E3 ligases that ubiquitylate histone H2A. To establish that BRCA1/BARD1 can ubiquitylate chromatin in cells independently of these confounding activities, we took advantage of the fact that its E3 function resides within a small region encompassing the RING domains of each protein. We developed an assay to measure the potential of the BRCA1/BARD1 heterodimeric RING complex, which was previously shown to adopt the same structure and have the same biochemical activity as the full-length BRCA1/BARD1 RING complex (Christensen et al., 2007), to ubiquitylate histone H2A at a defined site in the genome of U2OS 2-6-3 cells independently of its association with other proteins. The BARD1 RING (residues 26–126)-Gly_2_SerGly_2_-BRCA1 RING (residues 2–109) fusion protein (hereafter referred to as BDfBC) was fused to mCherry and Lac Repressor (LacI) sequences and transfected into U2OS 2-6-3 cells containing 200 copies of a transgene with a 256 × lac operator sequence (lacO) integrated at a single locus in the genome. Expression of BDfBC-mCherry-LacI in 2-6-3 cells coincided with the appearance of a single predominant mCherry nuclear focus that colocalized with conjugated ubiquitin (using FK2 antibody) or H2Aub (using E6C5 antibody; Figures 2A, 2B, and S2A), or after transfection of ubiquitin-GFP (data not shown). Recruitment of BDfBC also coincided with increased accumulation of H2Aub at the same site (Figures 2C, 2D, and S2B). Full-length BRCA1 protein, in which isoleucine 26 of its RING domain was mutated to alanine, is defective in E3 ligase activity in vitro (Figure S3A). Accordingly, site-specific H2Aub was not observed after expression of the “E3-ligase-dead” mutant BDfBC^I26A^-mCherry-LacI in vivo (Figures 2C and 2D). We concluded that the BRCA1/BARD1 RING domain complex can specifically ubiquitylate chromatin-associated H2A in vivo.

### BRCA1/BARD1 Ubiquitylates H2A at a Novel Site In Vitro and In Vivo

Next, we wished to identify the lysine residue on histone H2A that is modified by BRCA1/BARD1. Recently, RNF168 was shown to monoubiquitylate the N-terminal tail of histone H2A on lysines K13-15 (Mattiroli et al., 2012). However, this was not the case for BRCA1/BARD1, which efficiently ubiquitylated nucleosomes reconstituted with H2A deleted for its N-terminal tail (Figures S3B and S3C). In contrast, RING1B/MEL18 ubiquitylates histone H2A predominantly at lysine 119 in its C-terminal tail both in vitro and in vivo (Elderkin et al., 2007).

**Figure 3:**
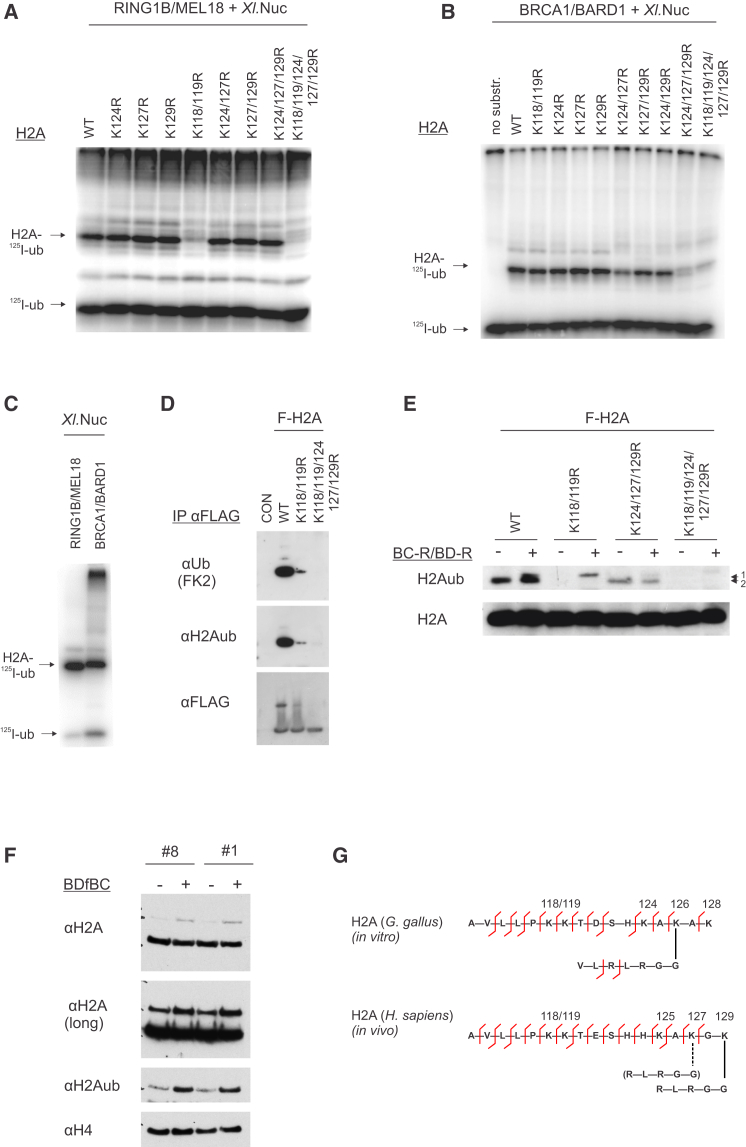
BRCA1/BARD1 Ubiquitylates K127-129 of Histone H2A In Vitro and In Vivo (A) Ubiquitylation of nucleosomes by RING1B/MEL18 is impaired by mutation of lysines 118 and 119 of histone H2A. (B) Ubiquitylation of nucleosomes by BRCA1/BARD1 is reduced for histone H2A mutated at lysines K124, K127, and K129. Small amounts of diubiquitylated histone were observed as indicated (^∗^). (C) Ubiquitylation of recombinant *Xenopus* nucleosomes by BRCA1/BARD1 and RING1B/MEL18, showing the difference in migration of the H2Aub product. (D) FLAG-H2A was stably expressed in DT40 cells and immunoprecipitated under denaturing conditions as described previously (Wang et al., 2004). Cells expressing wild-type or mutant H2A are indicated. Immunoprecipitated proteins were analyzed by western blot and probed with antibodies against FLAG, ubiquitin (FK2), and ubiquityl-H2A (E6C5). (E) DT40 cell lines expressing wild-type or mutant FLAG-H2A (K118/119R, K124/127/129, and K118/119/124/127/129) were transfected with the RING domains of BRCA1 and BARD1. Cells were harvested after 48 hr and histone proteins were isolated by acid extraction. FLAG-H2Aub was detected by western blot with anti-FLAG after separation by PAGE. H2Aub was detected after expression of the BRCA1 and BARD1 RING domains (band 1) and/or by endogenous E3 activity (band 2). (F) HEK293 cells expressing BDfBC-EGFP-NLS protein under the control of a Tet-responsive promoter was induced overnight with 1 μg/ml doxycycline. Nuclei were prepared from the cells and acid-extracted histones were separated on a 12% BisTris Novex gel and probed with the indicated antibodies. Increased H2Aub dependent on expression of BDfBC is indicated for two independent clones (#8 and #1). (G) Top, BRCA1/BARD1 ubiquitylates chicken histone H2A at lysine 126 (equivalent to K127 in humans) in nucleosomes. The modified peptide fragments identified by mass spectrometry are indicated. Data supporting this modification are provided in Figure S3E. Bottom, BDfBC expressed in HEK293 cells ubiquitylates human histone H2A at lysine K127-129. H2Aub was recovered from chromatin as described in the Experimental Procedures. The modified peptide fragments identified by mass spectrometry are indicated in the illustration. Data supporting this modification are provided in Figure S3F.

We tested recombinant *Xenopus laevis* nucleosomes containing H2A mutated in different lysines at its C-terminal tail. Whereas RING1B/MEL18 did not ubiquitylate nucleosomes reconstituted with mutant H2A K118-119R protein in vitro (Figure 3A; Elderkin et al., 2007), these same mutant nucleosomes were efficiently ubiquitylated by BRCA1/BARD1 (Figure 3B), indicating that it could modify residues other than K118 and K119. However, BRCA1-dependent ubiquitylation was markedly reduced with nucleosomes in which both K124 and K127 of H2A were mutated to arginine (H2A K124-127R) (Figure 3B). Ubiquitylation was further reduced in an H2A K124-127-129R mutant, suggesting that BRCA1/BARD1 ubiquitylates histone H2A in vitro at one or more of three residues (K124, K127, and K129). A direct comparison of H2Aub generated by RING1B/MEL18 and that produced by BRCA1/BARD1 in vitro revealed a small difference in migration after SDS-PAGE, providing further evidence that H2Aub produced by BRCA1/BARD1 in vitro occurred at a different lysine residue (Figure 3C).

To determine which of the lysines in the H2A C-terminal tail is the preferred site of ubiquitin conjugation by BRCA1/BARD1, we ubiquitylated chromatin purified from chicken erythrocytes in vitro and analyzed the products by mass spectrometry. We were able to identify ubiquitylation of the C-terminal peptide KAK (residues 126–128 of chicken H2A; Figures 3G, top, and S3E). Coupled with the detection of unmodified lysine 128, this suggested that K126 of chicken H2A (equivalent to K127 in *Xenopus* and human) might be the predominant residue for BRCA1/BARD1-dependent ubiquitin conjugation in vitro.

### BRCA1/BARD1 Ubiquitylates Human Histone H2A at K127 and K129 In Vivo

The most abundant form of H2A ubiquitylation in cells is K118-119. To determine the specific lysine in histone H2A that is ubiquitylated by BRCA1/BARD1 in vivo, we used DT40 cells stably expressing wild-type or mutant forms of FLAG-H2A. FLAG-H2A protein was immunoprecipitated with anti-FLAG, and western blots were probed with antibodies against H2Aub (E6C5), ubiquitin (FK2), or FLAG epitope (Figure 3D). This confirmed that mutant FLAG-H2A K118-119R was ubiquitylated, albeit at a greatly diminished level compared with wild-type H2A. Ubiquitylation was further reduced in cells expressing FLAG-H2A K118-119-125-127-129R, confirming ubiquitylation of H2A at its C-terminal tail in vivo (Figure 3D).

We next expressed the separate BRCA1 and BARD1 RING domains (BC-R/BD-R) in DT40 cells with different mutant FLAG-H2A proteins. In untransfected cells expressing wild-type FLAG-H2A, western blots revealed a single band corresponding to H2AubK119 (band 1; Figure 3E). Upon transfection with BC-R/BD-R, we observed a second, slower-migrating band (band 2) similar to that observed after ubiquitylation of H2A by BRCA1/BARD1 in vitro. Whereas band 1 was absent in cells expressing mutant FLAG-H2A K118-119R, band 2 was induced upon expression of BC-R/BD-R in these cells. Band 2 was not observed in cells expressing FLAG-H2A K124-127-129R mutant, suggesting that ubiquitylation required one or more of the three most C-terminal lysine residues of H2A. We observed a similar requirement for K125, K127, and K129 for ubiquitylation of FLAG-H2A K118-119R in HEK293 cells expressing the BDfBC RING domain complex (Figure S3D). Although these data highlight the importance of K127 for ubiquitylation of H2A, they did not establish which of the lysine residues (K125, K127, and K129) becomes conjugated to ubiquitin.

Given that H2Aub comprises only 5%–10% of all H2A in cells, the amount of H2Aub that was modified at lysine residues other than K118-119 was extremely low (Figures 3D and 3F). Expression of the RING fusion protein BDfBC in HEK293 cells significantly increased the cellular pool of H2Aub, indicating that H2A is an efficient substrate for this E3 even when it is not specifically directed to chromatin as a fusion protein with LacI (Figure 3F). By contrast, we observed no increase in H2Bub after expression of BDfBC (Figure S2B). Next, we purified chromatin-associated H2Aub from cells expressing BDfBC and analyzed it by mass spectrometry. The number and close proximity of lysine residues required us to digest H2Aub with pepsin at pH 1.3 rather than trypsin prior to mass spectrometry analysis. We found that although the majority of cellular H2Aub was modified on K118 or K119, expression of BDfBC coincided with the recovery of ubiquitylated peptide corresponding to the C-terminal residues GK (residues 128-129) of H2A (Figures 3G, bottom, and S3F). We also identified peptides consistent with low-level ubiquitin conjugation at K127 (Figure S3G). On the basis of these data, the primary acceptor for BRCA1 E3 activity is probably lysine 129 of histone H2A. However, lysine 127, which is important for efficient ubiquitylation of H2A by BRCA1/BARD1 in vitro and in vivo, may also be ubiquitylated.

## Discussion

To date, neither the substrate nor the function of the BRCA1/BARD1 ubiquitin ligase has been well established. We have established that BRCA1/BARD1 specifically ubiquitylates histone H2A in chromatin in vitro and in vivo. Our data support a role for BRCA1/BARD1 as a histone-H2A-specific ubiquitin ligase that ubiquitylates the C-terminal tail of H2A at the previously uncharacterized lysine residues K127-129.

Several pieces of evidence link BRCA1-dependent E3 activity with ubiquitylation of histones in chromatin. First, purified BRCA1/BARD1 ubiquitylates individual histone proteins in vitro, albeit with little specificity (Chen et al., 2002; Hashizume et al., 2001; Mallery et al., 2002). We show that the specificity of BRCA1/BARD1 for K127-129 of H2A is acquired only in a nucleosomal context, a characteristic that is shared with RING1B/MEL18 for ubiquitylation of H2Aub118-119 (Elderkin et al., 2007). Moreover, our data suggest that the ability to discriminate nucleosome substrate from free histone in vivo resides within the heterodimeric RING domains comprising amino acids 1–109 of BRCA1 and 1–126 of BARD1.

Second, the RING domains of BRCA1/BARD1 that confer its E3 activity are structurally related to the RING1B/MEL18 RING domain subunits of the histone-H2A-specific ubiquitin ligase PRC1 (Brzovic et al., 2006; Buchwald et al., 2006; Li et al., 2006). Of note, the basic patches on the surface of RING1B/BMI1, which have been shown to be involved in DNA binding of the E3-UbcH5 complex on nucleosomes (Bentley et al., 2011), are conserved in BRCA1, but not in BARD1. However, it is unclear whether this might affect the position of E3 relative to its nucleosome substrate.

We note that BRCA1/BARD1 and RING1B/MEL18 are heterodimeric E3 ligases (Brzovic et al., 2001; Buchwald et al., 2006; Li et al., 2006), whereas RNF168 is monomeric (Campbell et al., 2012) and therefore might interact with its chromatin substrate in a different manner (Mattiroli et al., 2012). Recent evidence suggests that ubiquitylation of histone H2A by RING1B/BMI1 and RNF168 is dependent on an acidic patch present on the exposed surface of nucleosomes. Expression of a peptide that interfered with binding to this acidic patch caused a reduction in DNA-damage-induced H2Aub by RNF168 and a concomitant failure to recruit BRCA1 at sites of DNA damage in vivo (Leung et al., 2014; Mattiroli et al., 2014). However, recruitment of BRCA1 at sites of DNA damage is dependent on RNF168-mediated ubiquitylation, and therefore no conclusion can be made regarding the effect on BRCA1 E3 ligase activity.

Third, our data support recent evidence indicating that small interfering RNA (siRNA)-mediated knockdown of BRCA1 results in derepression of satellite DNA with an accompanying loss of H2Aub in this region (Zhu et al., 2011). Our findings highlight the potential for BRCA1 to function directly in this process through ubiquitylation of histone H2A. This is consistent with the demonstration by Zhu et al. (2011) that repression of satellite DNA can be restored by expression of histone H2A protein fused at its C terminus with ubiquitin. Moreover, it suggests that although ubiquitylation of H2A on K127-129 is characteristic of BRCA1/BARD1 E3 function, the exact position of this ubiquitin in the C-terminal tail might not be critical for its role in repression of satellite DNA. It is possible that H2AubK127-129 and the more common H2Aub119 perform very similar functions in chromatin, and that BRCA1/BARD1 targets this modification to specific regions of chromatin.

BRCA1 has been linked to ubiquitin conjugates on chromatin at sites of DNA breaks. However, previous attempts to visualize the ubiquitylated products of BRCA1/BARD1 E3 activity at sites of DNA damage have been hampered by its complex recruitment to sites of DNA breaks involving a ubiquitin-mediated signaling pathway and several different ubiquitin ligases (Gospodinov and Herceg, 2013). Here, we show that site-specific targeting of the BRCA1/BARD1 RING domains is sufficient for local ubiquitylation of H2A in vivo. Mass spectrometry analysis confirmed that the biochemical activity and specificity of the BDfBC complex to ubiquitylate the C-terminal tail of H2A on lysines K127-129 in vivo reflect those of the full-length BRCA1/BARD1 complex in vitro. Moreover, using the “E3-dead” I26A mutant protein, we established that generation of H2Aub by BDfBC is specifically dependent on BRCA1 E3 activity.

Previous studies led us to believe that the position of ubiquitin within a nucleosome is more important for its function than the exact location of the modification within the histone tail. Indeed, it has been shown that ubiquitin fused to the very C-terminal amino acid of histone H2A contributes to the repression of satellite DNA. The exact role(s) of ubiquitin, however, is still enigmatic.

The contribution of BRCA1 E3 activity to the DNA damage response is unclear. Mice expressing the enzymatically dead mutant BRCA1(I26A) are no more tumor prone than those expressing wild-type protein (Shakya et al., 2011). On the other hand, murine embryonic stem cells expressing this BRCA1 I26A mutant protein have increased levels of genomic aberrations (Reid et al., 2008). It is possible that rather than promoting repair, the BRCA1 ubiquitin ligase functions as a negative regulator of double-strand break repair, as was shown recently for the ubiquitin-binding RAP80 complex (Coleman and Greenberg, 2011).

Although the role of monoubiquitylated H2A at DNA breaks is still unclear, our data establish the potential of BRCA1/BARD1 as a specific H2A ubiquitin ligase on nucleosome substrates. Moreover, the identification of a previously uncharacterized form of H2Aub that might be uniquely associated with the E3 activity of BRCA1 raises the possibility of generating antibodies directed against H2AubK127-129 for use as a diagnostic tool to identify biochemically active BRCA1.

## Experimental Procedures

### Cell Culture

DT40 chicken cells were propagated in standard media supplemented with RPMI (Invitrogen) at 37°C, 6% CO_2_. HeLa cells were cultured in Dulbecco’s modified Eagle’s medium (DMEM; Invitrogen) supplemented with 10% fetal calf serum. HEK293 FlpIn T-Rex cells (Invitrogen) were grown in DMEM containing 10% tetracycline-free fetal bovine serum.

### Microscopy

Cells were prepared as described in the Supplemental Experimental Procedures and visualized using an LSM510 confocal microscope (Leica).

### Ubiquitylation of Recombinant Nucleosomes and Chromatin

Recombinant *X. laevis* histones were expressed and purified from *E. coli* and reconstituted into nucleosomes as described in the Supplemental Experimental Procedures. The chicken histone octamers were a kind gift from Professor Daniela Rhodes and were purified from chicken erythrocyte nuclei as described previously (Thomas and Butler, 1980). For nucleosome ubiquitylation, chromatin or individual histones were incubated with 200 ng E1 (affinity or Boston Biochem), 200 ng UbcH5c (affinity or Boston Biochem), 1 μg ubiquitin (Sigma), 1 mM ATP and 0.1 μg purified E3 were in a reaction volume of 10 μl for 15 min, followed by addition of 10 μl of 0.5 μg ^125^I-ubiquitin, substrate and 1 mM in 1x ub buffer (Mallery et al., 2002). Reactions were stopped by addition of SDS buffer and applied for gel electrophoresis.

### Purification of Ubiquitylated Histones and Mass Spectrometry

H2Aub was isolated from cells by initial purification of H2A and H2B from chromatin in cells using the Histone Purification Kit (Active Motif). Histones were separated by PAGE using 12% Bis-Tris gel (Invitrogen) and MES buffer, and Coomassie-stained H2Aub bands were excised. In-gel digestion with pepsin (pH 1.3) was performed and peptides were analyzed by nanoLC-LTQ/Orbitrap in a data-dependent tandem mass spectrometry mode. For nucleosomes ubiquitylated in vitro, in-gel digestion was also performed with pepsin and analyzed by NextGen Bioscience.

## Author Contributions

R.K., D.L.M., C.L., and K.H. conceived and designed experiments. R.K., D.L.M., and C.L. performed experiments. J.T.J.H. performed mass spectrometry and analyzed the results. R.K. and K.H. wrote the manuscript.

## Supplemental Information

Document S1. Supplemental Experimental Procedures and Figures S1–S3

Document S2. Article plus Supplemental Information
