# Supplementary material for: BRCA1 Is a Histone-H2A-Specific Ubiquitin Ligase
Source: Cell Rep. 2014 Aug 14;8(4):999–1005. doi: 10.1016/j.celrep.2014.07.025 (PMC4382519; doi:10.1016/j.celrep.2014.07.025)
Supplement: Document S1. Supplemental Experimental Procedures and Figures S1–S3 [file mmc1.pdf]

Cell Reports, Volume *8*

Supplemental Information

BRCA1 Is a Histone-H2A-Specific Ubiquitin Ligase

Reinhard Kalb, Donna L. Mallery, Conor Larkin, Jeffrey T.J. Huang, and Kevin Hiom

Experimental Procedures

Cell culture

DT40 chicken cells were propagated in standard RPMI (Invitrogen) supplemented media at 37 °C, 6% CO_2. Transfections were carried out by electroporation as previously described (Mallery et al., 2002). Hela cells were cultured in DMEM (Invitrogen) supplemented with 10% FCS. HEK293 FlpIn T-Rex cells (Invitrogen) were grown in DMEM containing 10% tetracycline free FBS and 50 U/ml Penicillin, 50 µg/ml Streptomycin at 37˚C in 5% CO_2. BDfBC EGFP-NLS expressing cell lines were generated according to the manufacturer instructions. Individual clones were selected with 200 µg/ml Hygromycin B (Invitrogen). 2-6-3 cells containing the LacO array were cultured in DMEM supplemeneted with 10% FCS, 50U/ml Penicillin, 50 µg/ml Streptomycin, 100 µg/ml Hygromycin B at 37˚C in 5% CO_2.

BDfBC construct

The Bard1-Brca1 RING fusion construct was a gift from Rachel Klevit. The ORF was amplified and cloned into pSV2-Fok1 mCherryLacI (a gift from Roger Greenberg) digested with NheI and AgeI. Bard1-Brca1 RING – EGFP-NLS fusion construct was cloned by opening pSV2-BaBr mCherryLacI with AgeI and Kpn2I ligation of PCR amplified EGFP containing the SV40 NLS sequence to the C-terminus of EGFP to create BDfBC EGFP-NLS. BDfBC EGFP-NLS was then subcloned into the KpnI and NotI sites of pcDNA5/FRT/TO. I26A mutants of the Brca1 RING fusion were generated with the Quickchange Lightening Site directed mutagenesis kit. Flag-H2A

mutants were expressed from the pDest12.2 expression vector under the control of the CMV promoter. C-terminal tail mutants were generated by amplification of the H2A coding sequence using a reverse primer containing the relevant mutation(s). Sequences of the mutagenic primers are available on request. The mutant cDNA was then re-cloned back into pDest12.2 using standard molecular biology techniques. All plasmids were verified by sequencing.

Microscopy

2-6-3 cells were seeded at a density of 2.5 x 10^4 cells/cm^2 in a 12 well dish containing a coverslip. 24 hours later the cells were transfected with the relevant mCherryLacI constructs using XtermeGENE HP (Roche) according to manufacturers instructions. After 24 h cells were washed with PBS and then incubated with CKS buffer (10 mM PIPES pH7.0, 100 mM NaCl, 300 mM Sucrose, 3 mM $MgCl_2$) supplemented with 0.5% Triton-X-100 and protease inhibitors for 3 minutes at room temperature. Washed cells were fixed with 4% Paraformaldehyde for 10 minutes at room temperature, permeabilised in 0.5% Triton-X-100 in PBS for 3 x 5 minutes and blocked for 30 minutes at 37˚C in blocking buffer (1% BSA in PBS). Fixed cells were incubated with primary antibody at the relevant dilution in blocking buffer for 1 hour at 37˚C, washed with PBS and incubated with the appropriate secondary antibody, 250 µg/ml RNase A (Qiagen) and 1µM TO-PRO-3 for 30 minutes. After another wash in the dark the coverslips were mounted onto slides using ProLong GOLD (Invitrogen). Antibodies used were anti-H2Aub (Clone E6C5) (Millipore) 1:100 and FK2 (Enzo Life Science) 1:500. Appropriate AlexaFluor 488 secondary

antibodies (Invitrogen) were used at 1:1000. Images were captured on a Zeiss LSM510 confocal microscope using the 40x oil immersion objective (N/A 1.3). At least 100 mCherry foci were counted for each condition.

Proteins

C-terminal tagged FLAG/12xHis (FH) full-length human tagged BRCA1 and BARD1 cDNA was cloned into pDEST8 and expressed in Sf9 using the Bac-to-Bac baculovirus expression system (Invitrogen) and purified essentially as described in Mallery et al. (2002). In short, viruses with high expression levels for BRCA1-FH and BARD1-FH were co-infected in Sf9 insect cells for 48 h. Cells were harvested by centrifugation at 1200 rpm for 5 min. Cleared cell lysates were prepared from fresh cells by sonication in nickel lysis buffer (20 mM Tris–HCl pH 7.5, 500 mM KCl, 10% glycerol, 1% Triton X-100, 2 mM 2-mercapto ethanol) containing 5 mM imidazole and complete protease inhibitors (Roche), followed by ultracentrifugation at 35 000 rpm for 45 min. The lysate was loaded directly onto a 5 ml HiTrap Ni^{2+} chelating column (GE Healthcare) and washed with 10 column volumes of buffer A (20 mM Tris–HCl pH 7.5, 500 mM KCl, 10% glycerol, 0.1% Triton X-100, 2 mM 2-mercapto ethanol) followed by at least 10 column volumes of buffer A containing 60 mM imidazole. Proteins were eluted in 10 ml of buffer A containing 750 mM imidazole, and diluted 3.3 fold in HepA buffer (20 mM Tris-HCl (pH 7.4), 10% glycerol, 0.1% Triton-X 2 mM DTT) to give a final concentration of 150 mM KCl. The Ni^{2+} eluate was loaded onto a 1 ml heparin column (GE Healthcare) and washed with at least 10 cloumn columns HepA containing 150 mM KCl. BRCA1/BARD1 was eluted with a 150 mM -1 M KCl gradient. Peak protein

fractions, which eluted at approx. 800 mM KCl, were dialysed against storage buffer (25 mM Tris–HCl pH 7.5, 150 mM KCl, 10% glycerol and 2 mM DTT). Aliquots were frozen on liquid nitrogen and stored at -80°C.

BRCA1(I26A)-FH/BARD1-HA, Ring1B-FH/Mel18-HA, BC-R/BD-R (both FLAG, 12xHis tagged) were expressed as described for BRCA1/BARD1 and in (Elderkin et al., 2007). Purification was performed by Ni^{2+} affinity chromatography (Ni-NTA, Qiagen).

Reconstitution of recombinant *Xenopus laevis* nucleosomes

Histones (H2A, H2B, H3 and H4) were expressed in BL21(DE3)LysS and purified essentially as described by Luger and coworkers (Luger, 1996). Bacteria were lysed in 50 mM Tris-HCl pH7.4, 100 mM NaCl, 1 mM EDTA and proteinase inhibitors. Inclusion bodies were pelleted by centrifugation, washed several times w/o 1% Triton-X100 and solved in unfolding buffer (20 mM Tris-HCl pH7.4, 7 M guanidium HCl, 10 mM DTT). Equimolar amounts of core histones were mixed and dialysed against refold buffer (2M NaCl, 10 mM Tris-HCl pH 7.4, 5 mM 2-mercaptoethanol). Histone octamers were separated by gel filtration (Superdex S200, GE Healthcare). Equal amounts of octamers (recombinant *X. laevis* or purified from chicken erythrocyte chromatin) and 5S rDNA were used for nucleosome reconstitution via salt dialysis. Reconstituted nucleosomes were stored in TE buffer at 4 °C.

Nucleosome Aassembly

Nucleosomes were assembled using purified octamers (recombinant *X. laevis* or chicken) and polyglutamic acid (PGA; Sigma P4886) according to Stein et

al. (Stein et al., 1979). PGA and histones were mixed in a 2:1 ratio in 150 mM NaCl and incubated for 1-2 h at room temperature. Precipitates were removed by centrifugation and the supernatant (HP-mix) was stored at 4 °C. Different ratios of ^{32}P-body labeled DNA (Widom 601 sequence, 161 nt) and HP-mix were empirically tested to reveal the optimal conditions for nucleosome assembly. Nucleosomes were separated by native polyacrylamide gel electrophoresis (4.5% polyacrylamide, 0.25x TBE, 5% glycerol), assembled nucleosomes were cut out and eluted from the gel.

Binding assay

Approx. 10-40 fmol of gel eluted nucleosomes were incubated for 10 min on ice with BRCA1/BARD1 full-length proteins. The sample was then loaded on a native polyacrylamide gel (4.5% polyacrylamide, 0.4x TBE, 5% glycerol) and separated by gel electrophoresis. Migration of radiolabelled nucleosomes were detected by autoradiography of the dried gel (Phosphoimager, GE Healthcare).

Ubiquitylation Assays

The radioactive ubiquitylation assay was done as described previously (Mallery et al., 2002) with minor modifications. 200 ng E1 (affinity or Boston Biochem), 200 ng UbcH5c (affinity or Boston Biochem), 1 µg ubiquitin (Sigma), 1 mM ATP and 0.1 µg purified E3 were incubated in a reaction volume of 10 µl for 15 min, followed by additon of 10 µl of 0.5 µg ^{125}I-ubiquitin, substrate and 1 mM in 1x ub buffer. Reaction were stopped by addition of SDS buffer and applied for gel electrophoresis. ^{125}I-labeled products were

visualized using the Molecular Dynamics Typhoon Phosphorimager (GE Healthcare) and ImageQuaNT software. Non radioactive ubiquitylation was performed in a 20 µl reaction containing 50 ng E1, 50 ng UbcH5c, 0.5 µg ubiquitin and 0.8-1.0 µg 61*197 NRL nucleosome arrays/ native chromatin with 50 ng E3. Reaction was stopped by addition of SDS loading buffer.

Immunochemistry

For *in vivo* experiments chicken DT40 cell lines stably expressing FLAG-H2A (WT, K119R/K120R, K125R/K128R/K130R and K119R/K120R/K125R/K128R/K130R) were established. FLAG-H2A was bound to anti-FLAG M2 resign under denaturing conditons (Sigma) as described previously (Doil et al., 2009; Shiio and Eisenman, 2003). After elution with FLAG peptide the sample was separated via SDS PAGE and used for immunodetection. Pull down was checked by Coomassie staining. The ubiquitin E3 ligase activity of BRCA1/BARD1 on H2A was tested by transient co-expression of BRCA1 and BARD1 RING domains in indicated cell lines. 48h after transfection histones were isolated by acid extraction and immunoblotted using anti-FLAG antibody (M2, Sigma).

Histones of BDfBC cell lines were acid extracted. In short, cells were seeded at a density of 6 x 104 cells/cm^2 in a 10 cm dish. 24 h after transfection with plasmids carrying the ORF of Flag-H2A mutants the expression of BDfBC EGFP-NLS was induced with 5 µg/ml Dox. Cells were harvested 16-24 hours after induction, washed once with PBS and then resuspended in Buffer A (10 mM Hepes pH 7.0, 10mM KCl, 1.5Mm $MgCl_2$, 340 mM Sucrose, 10% Glycerol, 10mM NEM, 0.02% NaN_3 and 1 x Halt Protease Inhibitors (Thermo

Scientific) supplemented with 0.1% Triton-X-100). The samples were incubated on ice for 8 minutes then centrifuged at 1,300 x g for 5 minutes at 4˚C, washed once with Buffer A and then lysed in Buffer B (3mM EDTA, 0.2mM EGTA, 10 mM NEM, 0.02% NaN_3 and 1 x Halt Protease Inhibitors (Thermo Scientific)) on ice to release the chromatin from the nuclei. Samples were centrifuged at 1,700 x g for 5 minutes at 4˚C to separate the chromatin from the soluble nuclear fraction. The soluble nuclear fraction was retained to verify expression of the BDfBC EGFP-NLS. The resulting pellet was washed for 10 minutes with Buffer B supplemented with 350mM NaCl to remove loosely bound chromatin proteins. Following centrifugation at 1,700 x g for 5 minutes at 4˚C, 200 mM HCl was added to the resulting pellet and incubated overnight at 4˚C to extract the histones. Extracted histones were neutralized by addition of 1/5th the final volume of 2.5M Tris-Cl pH 8.0. The protein concentration was estimated using a Bradford assay. Equal amounts of protein were resolved on a 12% Bis-Tris gel ran using MES buffer. The proteins were transferred to a nitrocellulose membrane and probed with the appropriate antibodies. Antibodies used were Anti-Flag M2 (Sigma) 1:1000, Anti-GFP Clone B2 (Santa Cruz) 1:1000, Anti-H2B N-20 (Santa Cruz) 1:1000 and Anti-p84 clone 5E10 (GeneTex) 1:2000.

Mass spectrometry

H2Aub was purified from cells using Actif motif histone purification kit followed by gel purification of H2Aub after PAGE in a 12% Bis-Tris acrylamide gel using MES buffer. H2Aub was digested in gel and analyzed as described in Figure S3.

References

Brzovic, P.S., Rajagopal, P., Hoyt, D.W., King, M.C., and Klevit, R.E. (2001). Structure of a BRCA1-BARD1 heterodimeric RING-RING complex. Nature structural biology *8*, 833-837.

Buchwald, G., van der Stoop, P., Weichenrieder, O., Perrakis, A., van Lohuizen, M., and Sixma, T.K. (2006). Structure and E3-ligase activity of the Ring-Ring complex of polycomb proteins Bmi1 and Ring1b. EMBO J *25*, 2465-2474.

Doil, C., Mailand, N., Bekker-Jensen, S., Menard, P., Larsen, D.H., Pepperkok, R., Ellenberg, J., Panier, S., Durocher, D., Bartek, J., *et al.* (2009). RNF168 binds and amplifies ubiquitin conjugates on damaged chromosomes to allow accumulation of repair proteins. Cell *136*, 435-446.

Elderkin, S., Maertens, G.N., Endoh, M., Mallery, D.L., Morrice, N., Koseki, H., Peters, G., Brockdorff, N., and Hiom, K. (2007). A phosphorylated form of Mel-18 targets the Ring1B histone H2A ubiquitin ligase to chromatin. Mol Cell *28*, 107-120.

Huynh, V.A., Robinson, P.J., and Rhodes, D. (2005). A method for the in vitro reconstitution of a defined "30 nm" chromatin fibre containing stoichiometric amounts of the linker histone. J Mol Biol *345*, 957-968.

Lowary, P.T., and Widom, J. (1998). New DNA sequence rules for high affinity binding to histone octamer and sequence-directed nucleosome positioning. J Mol Biol *276*, 19-42.

Luger, K. (2003). Structure and dynamic behavior of nucleosomes. Curr Opin Genet Dev *13*, 127-135.

Mallery, D.L., Vandenberg, C.J., and Hiom, K. (2002). Activation of the E3 ligase function of the BRCA1/BARD1 complex by polyubiquitin chains. EMBO J *21*, 6755-6762.

Robinson, P.J., Fairall, L., Huynh, V.A., and Rhodes, D. (2006). EM measurements define the dimensions of the "30-nm" chromatin fiber: evidence for a compact, interdigitated structure. Proc Natl Acad Sci U S A *103*, 6506-6511.

Routh, A., Sandin, S., and Rhodes, D. (2008). Nucleosome repeat length and linker histone stoichiometry determine chromatin fiber structure. Proc Natl Acad Sci U S A *105*, 8872-8877.

Shiio, Y., and Eisenman, R.N. (2003). Histone sumoylation is associated with transcriptional repression. Proc Natl Acad Sci U S A *100*, 13225-13230.

Stein, A., Whitlock, J.P., Jr., and Bina, M. (1979). Acidic polypeptides can assemble both histones and chromatin in vitro at physiological ionic strength. Proc Natl Acad Sci U S A *76*, 5000-5004.

Thomas, J.O., and Butler, P.J. (1980). Size-dependence of a stable higher-order structure of chromatin. J Mol Biol *144*, 89-93.

Supplemental figures and legends

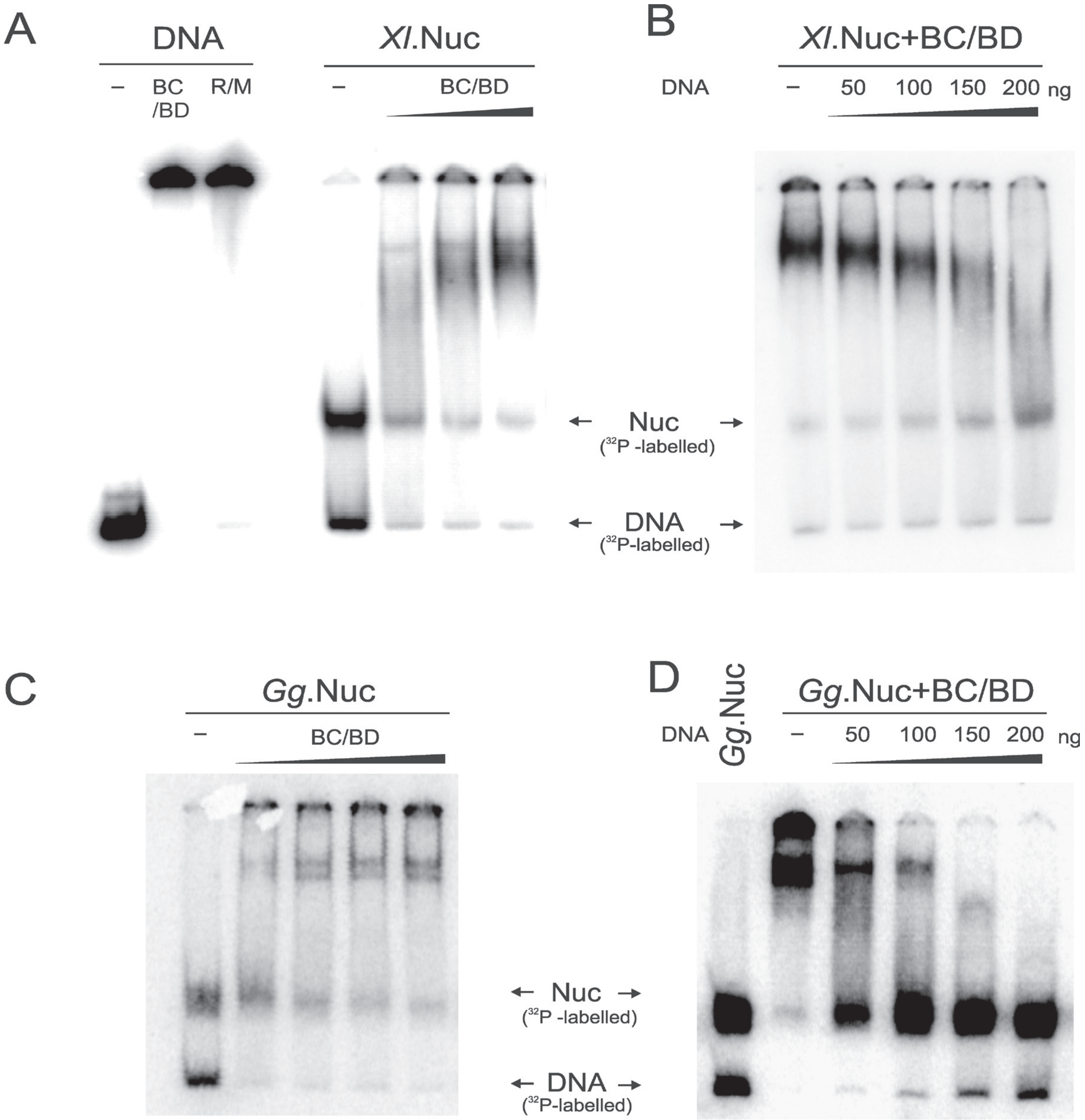

Figure S1 A-D. BRCA1/BARD1 binds to nucleosomes *in vitro*; Related to Figure 1. Recombinant full length BRCA1/BARD1 (BC/BD) and RING1B/MEL18 (R/M) were incubated with DNA, recombinant *Xenopus* nucleosomes (**A** and **B**) or nucleosomes reconstituted with chicken erythrocyte octamers (**C** and **D**) and analyzed in an Electrophoretic Mobility Shift Assay (EMSA). For nucleosome reconstitution radiolabelled “601” DNA (161 nt) (Lowary and Widom, 1998) was used. Per reaction approx. 10-40 fmol of gel eluted nucleosomes were incubated with 0.5, 1.0, 1.5 or 2.0 µg of full-length recombinant BRCA1/BARD1 or RING1B/MEL18 in binding buffer (50 mM Tris-HCl pH 7.4, 2.5 mM $MgCl_2$, 0.5 mM DTT). Reactions were loaded on a native TBE polyacrylamide gel (4.5% polyacrylamide, 0.25x TBE). Gels were dried and radiolabelled products were visualized using a Typhoon phosphoimager (GE Healthcare). Competition assays were performed as in the binding reactions described above with 2.0 µg BRCA1/BARD1, followed by the addition of indicated amounts of unlabelled competitor 601 DNA (**B** and **D**).

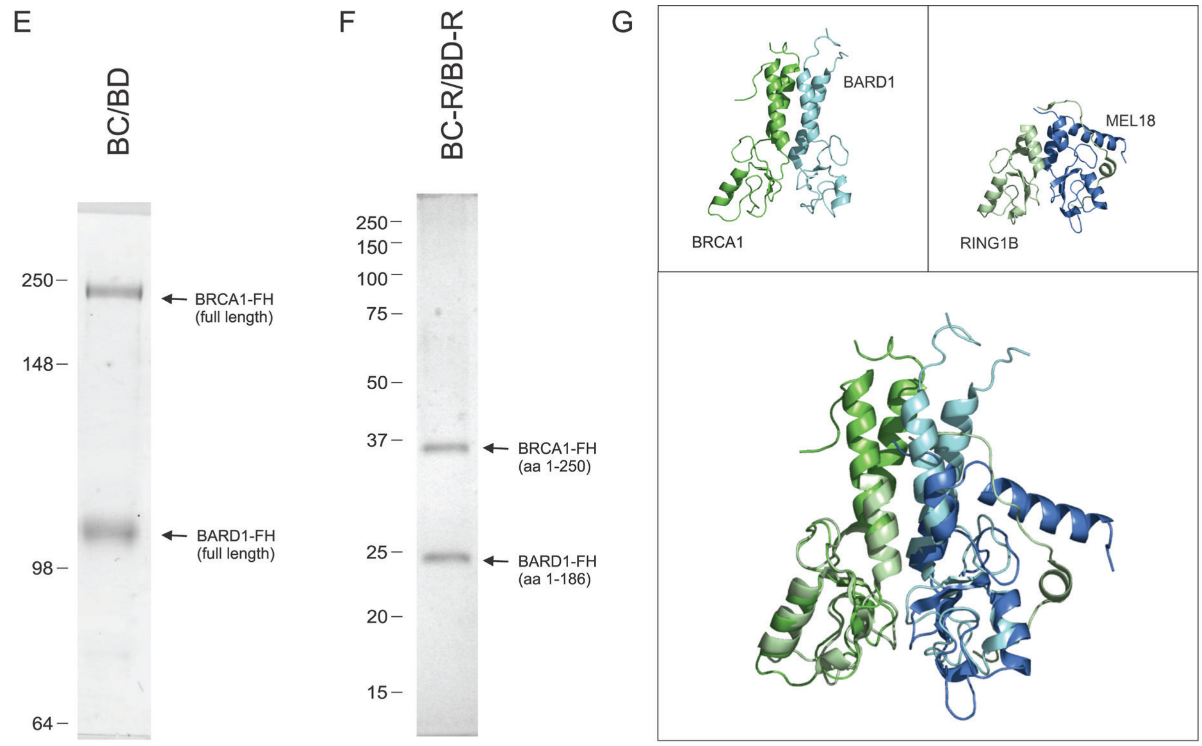

Figure S1 E-G: BRCA1/BARD1 RING domains share structural and biochemical properties with RING1B/MEL18; Related to Figure 1. (**E** and **F**) Polyacrylamide gel of recombinant full-length BRCA1/BARD1 (BC/BD) and RING domain complexes comprising residues 1-250 of BRCA1 and 1-186 BARD1 purified from Sf9 cells are shown. (**G**) Structure of the heterodimeric RING complexes formed by BRCA1/BARD1 (top left) and RING1B/MEL18 (top right) based on the diffraction data with the references 2CKL (Buchwald et al., 2006) and 1JM7 (Brzovic et al., 2001). The RING domain structure of MEL18 was predicted using ESyPred3D and visualized in PyMOL (Schroedinger) based on the high degree of identity with its orthologue Bmi1. The structures of BRCA1/BARD1 RING domains superimposed on that of RING1B/MEL18 (bottom) are depicted.

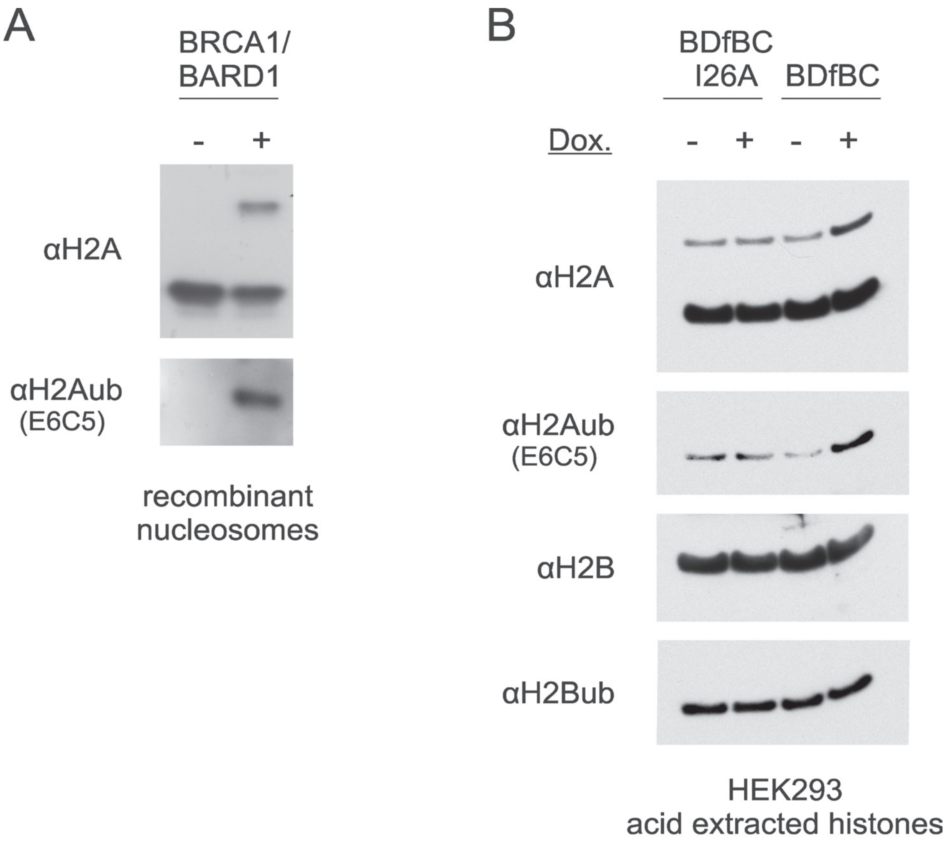

Figure S2: BRCA1/BARD1 ubiquitylates H2A *in vitro* and *in vivo*; Related to Figure 2. (**A**) Full length BRCA1/BARD1 ubiquitylates H2A in recombinant nucleosomes *in vitro*. The appearance of a 24 kDa band in an immunoblot using an specific anti-H2A antibody indicates ubiquitylated H2A. Notably, the anti-H2Aub antibody E6C5 detects H2A ubiquitylated by BRCA1/BARD1 in immunblots. (**B**) BDfBC (wt) and BDfBC I26A (mutant) were expressed in stable inducible HEK293 cell lines. 16-24 h after induction of expression with Dox (5µg/ml) cells were harvested. Acid extracted histones were resolved by SDS polyacrylamide gel electrophoresis, transferred to a nitrocellulose membrane and probed with the indicated antibodies.

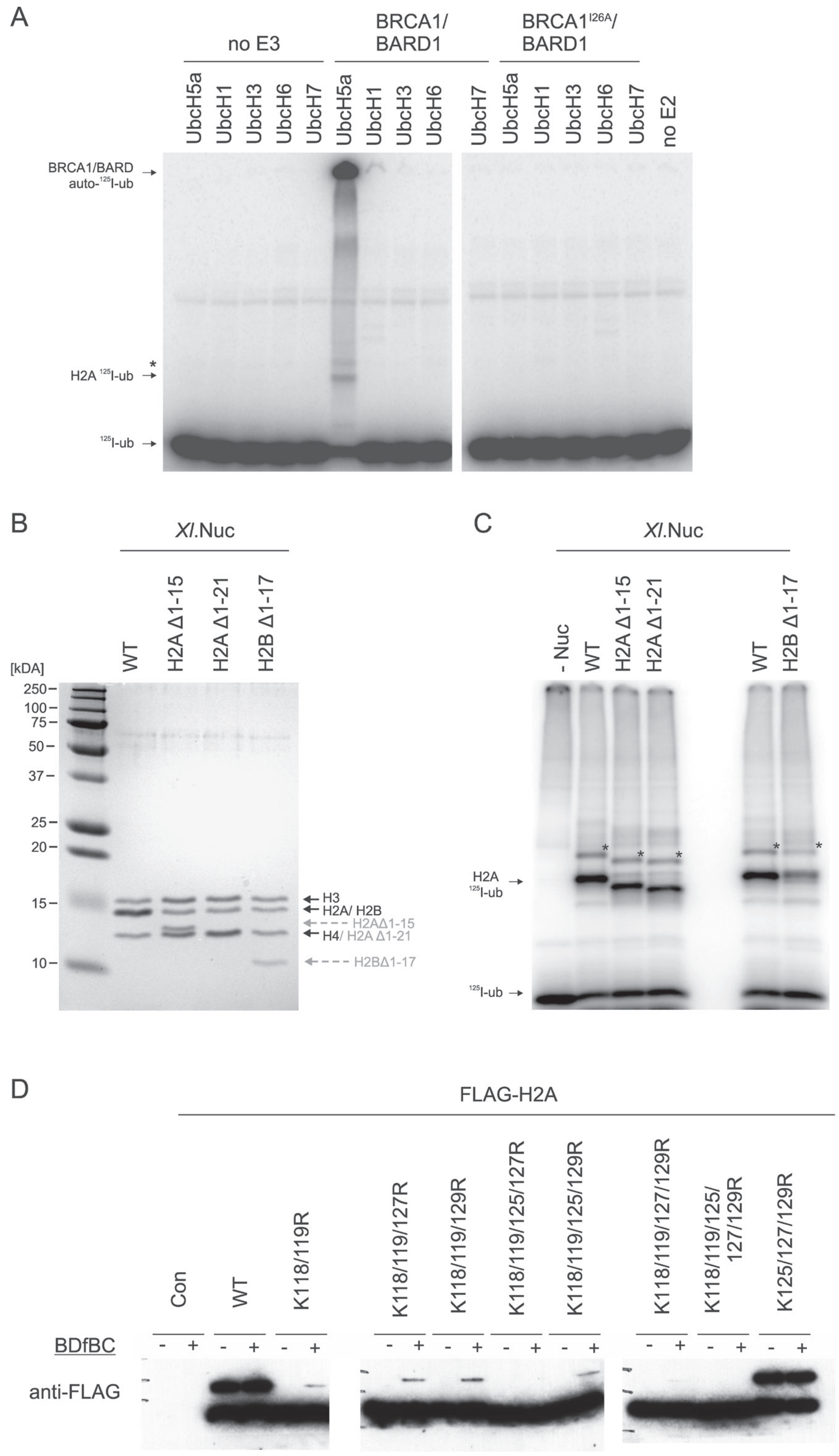

Figure S3 A-D: BRCA1/BARD1 ubiquitylates H2A at lysines K127-K129, Related to Figure 3. (**A**) BRCA1/BARD1 ubiquitylates H2A specifically with UbcH5 as the E2 enzyme as indicacted by the appearance of band representing H2A linked with ^{125}I-labeled ubiquitin. A point mutation in BRCA1 (I26A) which disrupts the binding site for UbcH5 is deficient for H2A ubiquitylation. (**B**) Coomassie stained SDS-PAGE of recombinant nucleosomes containing histones H2A and H2B with small N-terminal deletions as indicated (H2A Δaa1-15 and Δaa1-21, H2B Δaa1-17). (**C**) *In vitro* ubiquitinylation of recombinant nucleosomes with ^{125}I-labeled ubiquitin. Nucleosomes with deletions in the N-terminal tail of H2A or H2B are indicated. (**D**) HEK293 FlpIn T-Rex cell expressing Tet-inducible BDfBC, were transfected with plasmid expressing FLAG-tagged histone

H2A mutants as indicated. Chromatin was isolated from cells following induction of BDfBC expression by Doxycycline and histones purified by acid precipitation and separated by PAGE as described in Experimental procedures. Ubiquitinylation status of the mutant Histones was monitored by western blot using an anti-FLAG antibody.

E

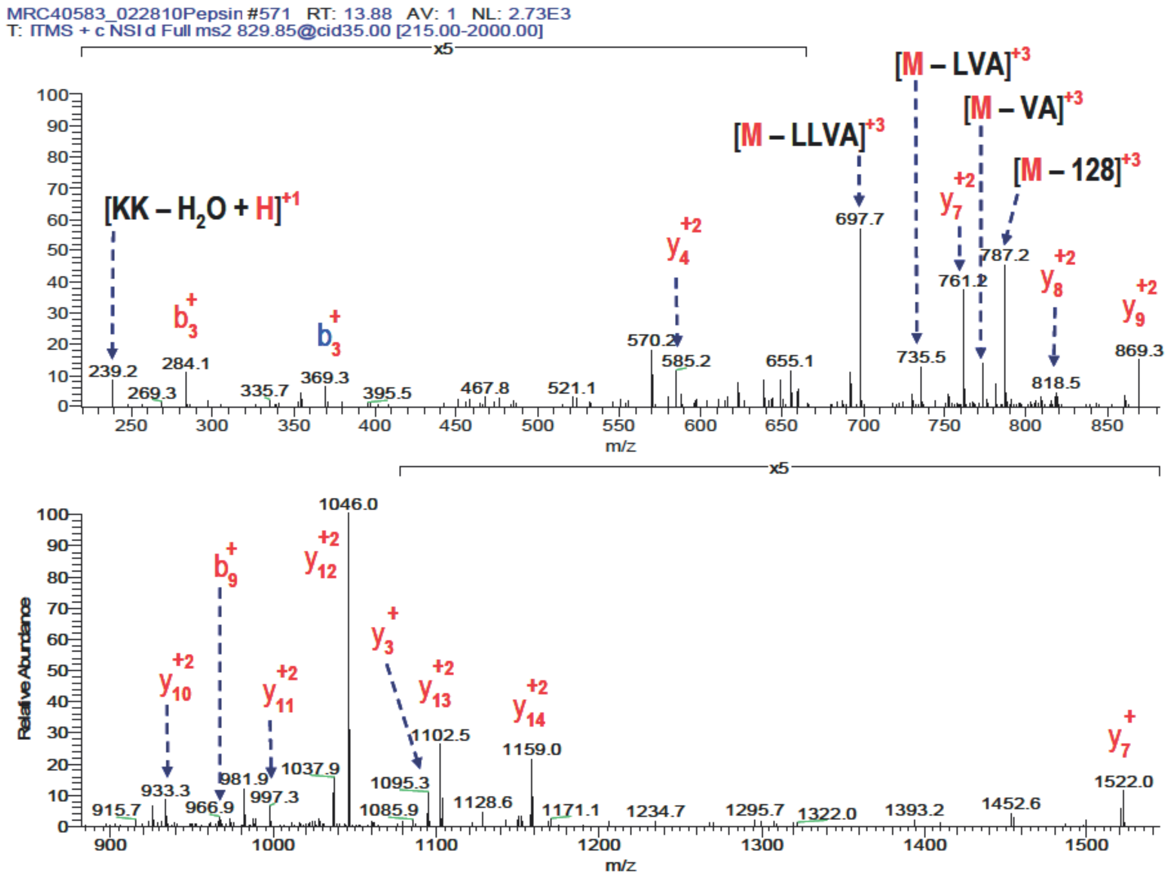
MRC40583_022810Pepsin #571 RT: 13.88 AV: 1 NL: 2.73E3
T: ITMS + c NSI d Full ms2 829.85@cid35.00 [215.00-2000.00]
x5
[KK – H2O + H]+1
[M – LLVA]+3
[M – LVA]+3
[M – VA]+3
[M – 128]+3
697.7
787.2
761.2
735.5
869.3
818.5
570.2
585.2
655.1
239.2
284.1
269.3
335.7
369.3
395.5
467.8
521.1
m/z
Relative Abundance
1046.0
1102.5
1159.0
1522.0
933.3
981.9
966.9
997.3
1037.9
1095.3
1085.9
1128.6
1171.1
1234.7
1295.7
1322.0
1393.2
1452.6
915.7

F

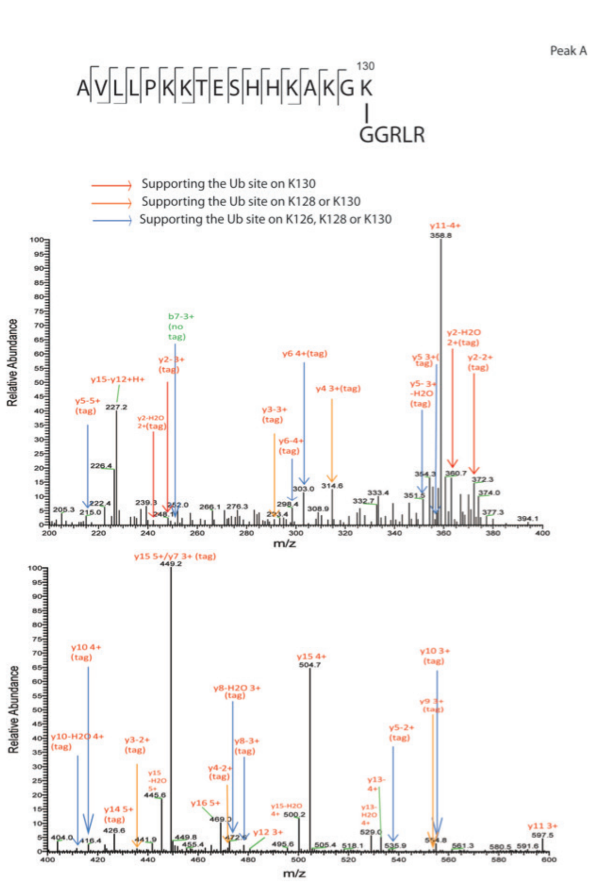
Peak A
A V L L P K K T E S H H K A K G K
130
GGRLR
Supporting the Ub site on K130
Supporting the Ub site on K128 or K130
Supporting the Ub site on K126, K128 or K130
Relative Abundance
m/z

G

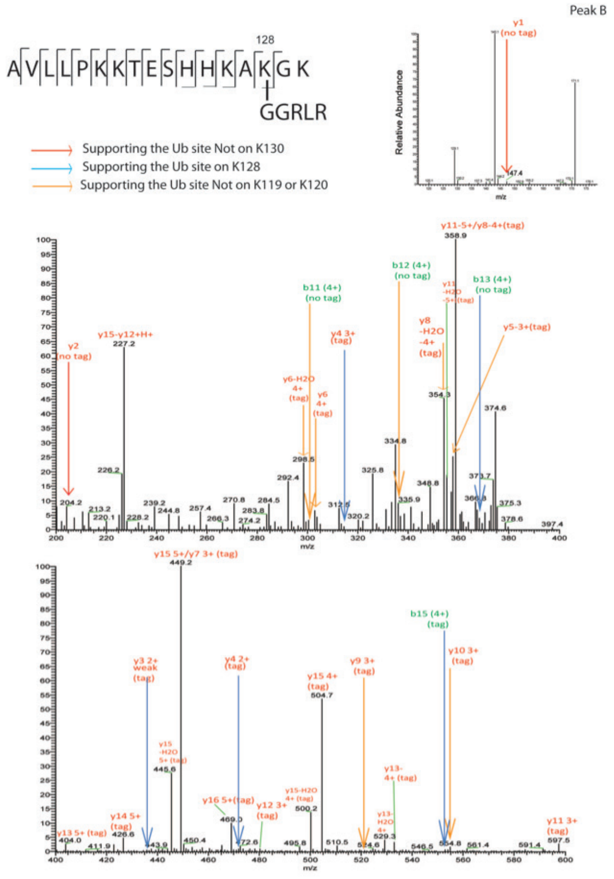
Peak B
A V L L P K K T E S H H K A K G K
128
GGRLR
Supporting the Ub site Not on K130
Supporting the Ub site on K128
Supporting the Ub site Not on K119 or K120
Relative Abundance
m/z

Fig. S3 E-G: H2A is specifically modified by BRCA1/BARD1 at lysines 127-129, Related to Figure 3G

(**E**) MS/MS data indicating that K127 of the H2A peptide AVLLPKKTDSHKAKAK is ubiquitinated by BRCA1/BARD1. Chicken chromatin was ubiquitylated by BC-R/BD-R *in vitro*, separated by SDS PAGE. Coomassie-stained H2A and H2Aub bands were excised, digest in-gel with chymotrypsin and pepsin and analysed by mass spectrometry (NextGen Bioscience, Ann Arbor). The peptide depicted was obtained by digestion with pepsin. Comparison of the spectra for unmodified vs ubiquitylated H2A peptide showed that VLRLRGG (ubiquitin) was bound to AVLLPKKTDSHKAKAK (H2A) at lysine 127 (the penultimate lysine) in two independent samples. Five charge states from +3 to +7, m/z = 829.5163 (+3), m/z = 622.3885 (+4), m/z = 498.1120 (+5), m/z = 415.2615 (+6), and m/z = 356.0827 (+7) were observed for this peptide complex. MS/MS data acquired on all these charge states are typical fragmentation patterns for highly protonated peptides and contained both a common set of product ions as well as ions unique to a particular charge state. The complete MS report including y1 is available on request. (**F**, **G**) MS/MS data indicating that K127 and K129 of the H2A peptide AVLLPKKTDSHKAKAK is ubiquitinated by BDfBC *in vivo*. The expression of BDfBC in HEK293 FlpIn T-Rex cell was induced by the addition of Doxycycline for 24 hours. Histones were purified from the cells and separated by PAGE. The region of the gel corresponding to the size of H2Aub was excised, in gel digested with pepsin (pH1.3) and analysed by nanoLC-LTQ/Orbitrap in a data dependent MS/MS mode. Peaks were observed that are consistent with the modification of AVLLPKKTESHHKAKGK peptide with ubiquitin tag (RLRGG). MS/MS spectra for these peaks generated by CID are annotated manually. The MS/MS spectrum shown in e suggests the modification of the K129 residue with RLRGG. A wide range of y ion series depicted by blue and orange arrows indicates that the modification locates at K127 or K129. The four y2 ions (y2(2+), y2(3+), y2-H2O(2+), y2-H2O(3+), red cursors) supports that the modification site is at K129. The MS/MS spectrum in shows a modification of the K127 residue with RLRGG supported by both y and b ion series. The signals matched to b11, b12, b13, b15, y3, y4, y5 and y8 ions suggest that the modification is located at either K127 or K129. In addition, the presence of y1 ion at 147.4, y2 ion at 204.2 and the absence of four y2 ions observed in E suggest that the modification site is not located at K129. K127 is therefore the likely site of modification in **G**. The indicated amino acid positons in E and F refer to the translated cDNA sequence including the post-translationally removed initial methionine.
