# Supplementary material for: BRCA1 Is a Histone-H2A-Specific Ubiquitin Ligase
Source: Cell Rep. 2014 Aug 14;8(4):999–1005. doi: 10.1016/j.celrep.2014.07.025 (PMC4382519; doi:10.1016/j.celrep.2014.07.025)
Supplement: Document S2. Article plus Supplemental Information [file mmc2.pdf]

Cell Reports
Report

OPEN ACCESS
CellPress

# BRCA1 Is a Histone-H2A-Specific Ubiquitin Ligase

Reinhard Kalb,[1,2,5] Donna L. Mallery,[1,5] Conor Larkin,[3] Jeffrey T.J. Huang,[4] and Kevin Hiom[1,3,*]
[1]Division of Protein Nucleic Acid Chemistry, MRC Laboratory of Molecular Biology, Francis Crick Avenue, Cambridge Biomedical Campus, Cambridge CB2 0QH, UK
[2]Department of Chromatin Research, MPI of Biochemistry, Am Klopferspitz 18, 82152 Martinsried, Germany
[3]Division of Cancer Research, Medical Research Institute, Ninewells Hospital & Medical School, Dundee DD1 9SY, Scotland
[4]Biomarker and Drug Analysis Core Facility, Medical Research Institute, Ninewells Hospital & Medical School, Dundee DD1 9SY, Scotland
[5]Co-first author
*Correspondence: k.hiom@dundee.ac.uk
http://dx.doi.org/10.1016/j.celrep.2014.07.025
This is an open access article under the CC BY license (http://creativecommons.org/licenses/by/3.0/).

## SUMMARY

**The RING domain proteins BRCA1 and BARD1 comprise a heterodimeric ubiquitin (E3) ligase that is required for the accumulation of ubiquitin conjugates at sites of DNA damage and for silencing at DNA satellite repeat regions. Despite its links to chromatin, the substrate and underlying function of the BRCA1/BARD1 ubiquitin ligase remain unclear. Here, we show that BRCA1/BARD1 specifically ubiquitylates histone H2A in its C-terminal tail on lysines 127 and 129 in vitro and in vivo. The specificity for K127-129 is acquired only when H2A is within a nucleosomal context. Moreover, site-specific targeting of the BRCA1/BARD1 RING domains to chromatin is sufficient for H2Aub foci formation in vivo. Our data establish BRCA1/BARD1 as a histone-H2A-specific E3 ligase, helping to explain its localization and activities on chromatin in cells.**

## INTRODUCTION

Breast cancer-associated protein 1 (BRCA1) is a key mediator in the DNA damage response, which is linked to a wide range of functions that serve to maintain genomic stability. Cellular BRCA1 forms a heterodimer with BRCA1-associated RING domain 1 (BARD1) (Wu et al., 1996), which promotes the repair of double-stranded DNA breaks through homologous recombination (Moynahan and Jasin, 2010) and contributes to the DNA-damage-induced G2/M checkpoint (Xu et al., 2001). Loss of BRCA1 function in cells results in hypersensitivity to DNA damage and accumulation of chromosomal aberrations associated with the development of cancer (Venkitaraman, 2001).

In vitro studies have shown that the RING domains of BRCA1/BARD1 possess a ubiquitin ligase (E3) function (Ruffner et al., 2001), but a bona fide substrate for this activity is still lacking. During replication and after treatment with agents that damage DNA, BRCA1 and BARD1 colocalize in discreet nuclear foci with ubiquitin conjugates (Morris and Solomon, 2004). However, it is not known whether these conjugates are a product of BRCA1/BARD1 E3 activity or arise through the function of several other E3 proteins that also colocalize at sites of DNA double-strand breaks as part of a ubiquitin-mediated DNA-damage-signaling pathway (Doil et al., 2009; Mattiroli et al., 2012; Stewart et al., 2009).

Recently, Zhu et al. (2011) reported that defects in BRCA1 E3 function are linked with a derepression of satellite DNA that is accompanied by decompaction of chromatin and reduced levels of ubiquitylated histone H2A (H2Aub). Moreover, these phenotypes can be reversed by exogenous expression of histone H2A protein fused to ubiquitin. However, a direct role for BRCA1/BARD1-dependent ubiquitylation of histones was not established.

We investigated the interaction of BRCA1/BARD1 with chromatin and found that it involves a highly specific histone H2A-ubiquitin ligase that modifies previously uncharacterized lysines in the C-terminal tail of H2A. We discuss these observations in light of the known biological functions of BRCA1.

## RESULTS

### BRCA1/BARD1 Ubiquitylates H2A in Nucleosomes

Although BRCA1 binds to DNA in a sequence-independent manner in vitro (Paull et al., 2001), in cells, it is most commonly found at DNA breaks associated with chromatin. Accordingly, we found that purified recombinant BRCA1/BARD1 bound nucleosome substrates in an electrophoretic mobility shift assay (EMSA) (Figures S1A–S1F). The binding was dynamic, as complexes were competed away by addition of unlabeled DNA (Figure S1D). This binding, along with the known structural similarities between the RING domains of BRCA1/BARD1 and those of the histone-H2A-specific ubiquitin ligase complex Polycomb repressive complex 1 (PRC1) (Buchwald et al., 2006; Figure S1G), prompted us to investigate whether BRCA1/BARD1 ubiquitylates nucleosomal histone proteins.

We examined BRCA1/BARD1 E3 activity on individual histone proteins and reconstituted nucleosome substrates in vitro and compared it with the activity of the RING1B/MEL-18 subunits of PRC1 (Figures 1A and 1B). As reported previously, RING1B/MEL18 monoubiquitylated individual histone proteins with similar efficiency in vitro, but ubiquitylated nucleosome substrates specifically on K118-119 of H2A (Figure 1A). This reflects the known cellular specificity of PRC1 for K118-119 of H2A

CrossMark

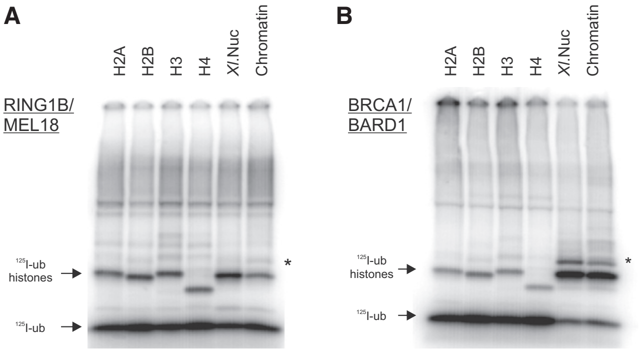


**Figure 1. BRCA1/BARD1 Specifically Ubiquitylates Histone H2A in Nucleosomes**

(A and B) Ubiquitylation of recombinant *Xenopus laevis* histones (H2A, H2B, H3, and H4), reconstituted nucleosomes, and chromatin isolated from HeLa cells by RING1B/MEL18 (A) or BRCA1/BARD1 (B) ubiquitin ligases. $^{125}$I-labeled ubiquitin is covalently linked to its substrate and detected after SDS-PAGE. Specificity for a single histone occurs only within a nucleosomal context. Small amounts of diubiquitylated histone were observed as indicated (*). See also Figure S1.

(Elderkin et al., 2007; Wang et al., 2004). BRCA1/BARD1 acted similarly, ubiquitylating individual H2A, H2B, H3, and H4 proteins in vitro without any apparent preference (Mallery et al., 2002; Figure 1B). However, when BRCA1/BARD1 was incubated with recombinant nucleosomes or native chromatin, only H2A was ubiquitylated (Figure 1B). We concluded that BRCA1/BARD1 E3 activity resembles that of PRC1 in that it acquires specificity for histone H2A as a substrate when the latter is incorporated into nucleosomes.

## BRCA1/BARD1 RING Domains Ubiquitylate H2A In Vivo

The recruitment of BRCA1/BARD1 to sites of DNA damage is dependent on the activity of several other E3 ligases that ubiquitylate histone H2A. To establish that BRCA1/BARD1 can ubiquitylate chromatin in cells independently of these confounding activities, we took advantage of the fact that its E3 function resides within a small region encompassing the RING domains of each protein. We developed an assay to measure the potential of the BRCA1/BARD1 heterodimeric RING complex, which was previously shown to adopt the same structure and have the same biochemical activity as the full-length BRCA1/BARD1 RING complex (Christensen et al., 2007), to ubiquitylate histone H2A at a defined site in the genome of U2OS 2-6-3 cells independently of its association with other proteins. The BARD1 RING (residues 26–126)-Gly$_2$SerGly$_2$-BRCA1 RING (residues 2–109) fusion protein (hereafter referred to as BDfBC) was fused to mCherry and Lac Repressor (LacI) sequences and transfected into U2OS 2-6-3 cells containing 200 copies of a transgene with a 256 × lac operator sequence (lacO) integrated at a single locus in the genome. Expression of BDfBC-mCherry-LacI in 2-6-3 cells coincided with the appearance of a single predominant mCherry nuclear focus that colocalized with conjugated ubiquitin (using FK2 antibody) or H2Aub (using E6C5 antibody; Figures 2A, 2B, and S2A), or after transfection of ubiquitin-GFP (data not shown). Recruitment of BDfBC also coincided with increased accumulation of H2Aub at the same site (Figures 2C, 2D, and S2B). Full-length BRCA1 protein, in which isoleucine 26 of its RING domain was mutated to alanine, is defective in E3 ligase activity in vitro (Figure S3A). Accordingly, site-specific H2Aub was not observed after expression of the "E3-ligase-dead" mutant BDfBC$^{I26A}$-mCherry-LacI in vivo (Figures 2C and 2D). We concluded that the BRCA1/BARD1 RING domain complex can specifically ubiquitylate chromatin-associated H2A in vivo.

## BRCA1/BARD1 Ubiquitylates H2A at a Novel Site In Vitro and In Vivo

Next, we wished to identify the lysine residue on histone H2A that is modified by BRCA1/BARD1. Recently, RNF168 was shown to monoubiquitylate the N-terminal tail of histone H2A on lysines K13-15 (Mattiroli et al., 2012). However, this was not the case for BRCA1/BARD1, which efficiently ubiquitylated nucleosomes reconstituted with H2A deleted for its N-terminal tail (Figures S3B and S3C). In contrast, RING1B/MEL18 ubiquitylates histone H2A predominantly at lysine 119 in its C-terminal tail both in vitro and in vivo (Elderkin et al., 2007).

We tested recombinant *Xenopus laevis* nucleosomes containing H2A mutated in different lysines at its C-terminal tail. Whereas RING1B/MEL18 did not ubiquitylate nucleosomes reconstituted with mutant H2A K118-119R protein in vitro (Figure 3A; Elderkin et al., 2007), these same mutant nucleosomes were efficiently ubiquitylated by BRCA1/BARD1 (Figure 3B), indicating that it could modify residues other than K118 and K119. However, BRCA1-dependent ubiquitylation was markedly reduced with nucleosomes in which both K124 and K127 of H2A were mutated to arginine (H2A K124-127R) (Figure 3B). Ubiquitylation was further reduced in an H2A K124-127-129R mutant, suggesting that BRCA1/BARD1 ubiquitylates histone H2A in vitro at one or more of three residues (K124, K127, and K129). A direct comparison of H2Aub generated by RING1B/MEL18 and that produced by BRCA1/BARD1 in vitro revealed a small difference in migration after SDS-PAGE, providing further evidence that H2Aub produced by BRCA1/BARD1 in vitro occurred at a different lysine residue (Figure 3C).

To determine which of the lysines in the H2A C-terminal tail is the preferred site of ubiquitin conjugation by BRCA1/BARD1, we ubiquitylated chromatin purified from chicken erythrocytes in vitro and analyzed the products by mass spectrometry. We were able to identify ubiquitylation of the C-terminal peptide KAK (residues 126–128 of chicken H2A; Figures 3G, top, and S3E). Coupled with the detection of unmodified lysine 128, this suggested that K126 of chicken H2A (equivalent to K127 in *Xenopus* and human) might be the predominant residue for BRCA1/BARD1-dependent ubiquitin conjugation in vitro.

## BRCA1/BARD1 Ubiquitylates Human Histone H2A at K127 and K129 In Vivo

The most abundant form of H2A ubiquitylation in cells is K118-119. To determine the specific lysine in histone H2A that is ubiquitylated by BRCA1/BARD1 in vivo, we used DT40 cells stably expressing wild-type or mutant forms of FLAG-H2A. FLAG-H2A protein was immunoprecipitated with anti-FLAG, and western blots were probed with antibodies against H2Aub (E6C5),

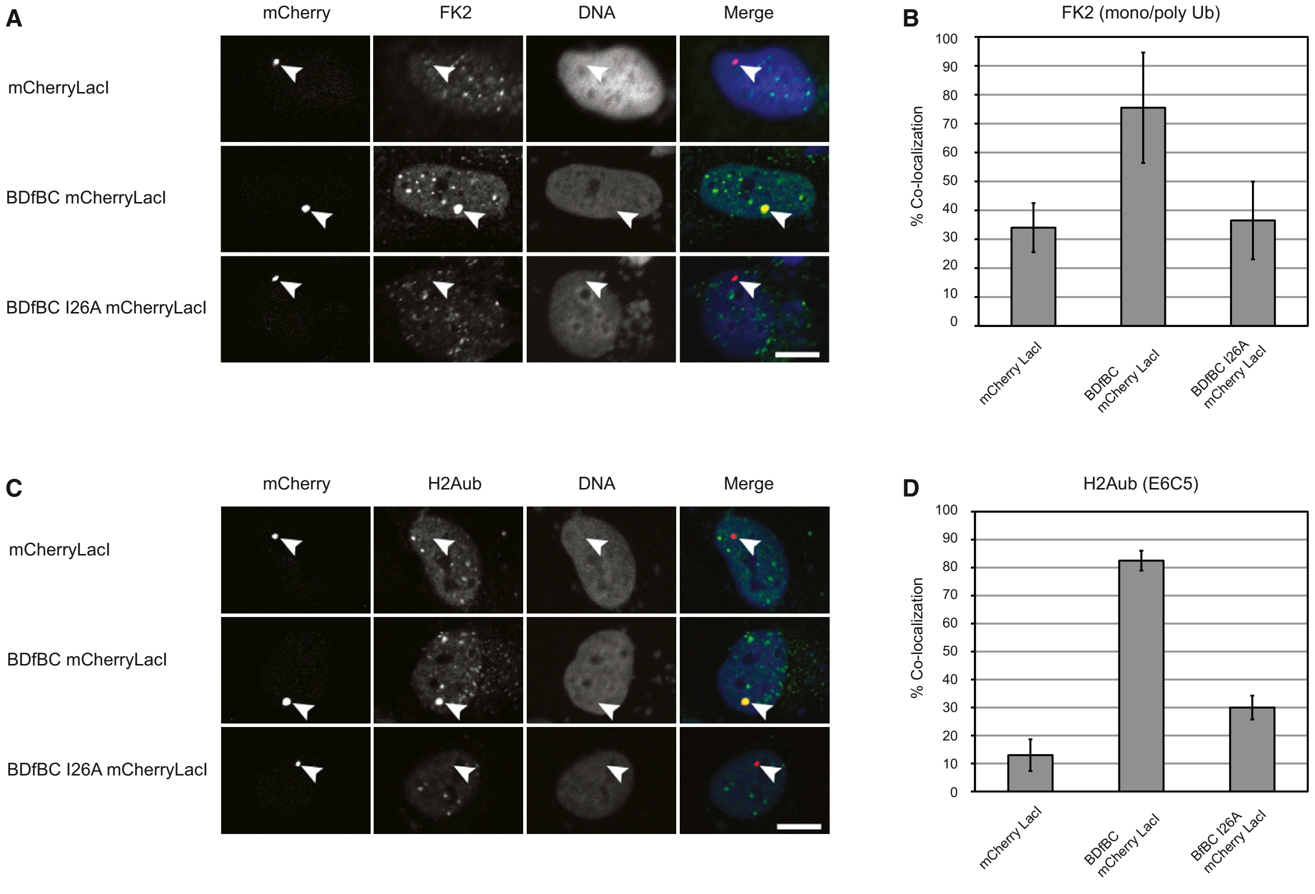


**Figure 2. Ubiquitylation of Histone H2A by BRCA1/BARD1 E3 Activity In Vivo**

(A and C) Site-specific recruitment of the BRCA1/BARD1 E3 to a single genomic location in HEK293 2-6-3 cells results in ubiquitylation of histone H2A. U2OS 2-6-3 cells containing 200 tandem copies of a 256 LacO sequence integrated at a specific site were transiently transfected with plasmids expressing mCherryLacI, BDfBC-mCherryLacI, or mutant BDfBC-I26A-mCherryLacI.

(A and B) Cells were stained with antibodies against H2Aub (E6C5; A) and scored for colocalization with mCherry (B).

(C and D) Cells were stained with antibodies against ubiquitin (FK2; C) and scored for colocalization with mCherry (D).

Representative images are shown. Values represent the mean from two independent experiments (n = 100). Error bars represent 1 SD. Scale bar represents 10 μm. See also Figure S2.

ubiquitin (FK2), or FLAG epitope (Figure 3D). This confirmed that mutant FLAG-H2A K118-119R was ubiquitylated, albeit at a greatly diminished level compared with wild-type H2A. Ubiquitylation was further reduced in cells expressing FLAG-H2A K118-119-125-127-129R, confirming ubiquitylation of H2A at its C-terminal tail in vivo (Figure 3D).

We next expressed the separate BRCA1 and BARD1 RING domains (BC-R/BD-R) in DT40 cells with different mutant FLAG-H2A proteins. In untransfected cells expressing wild-type FLAG-H2A, western blots revealed a single band corresponding to H2AubK119 (band 1; Figure 3E). Upon transfection with BC-R/BD-R, we observed a second, slower-migrating band (band 2) similar to that observed after ubiquitylation of H2A by BRCA1/BARD1 in vitro. Whereas band 1 was absent in cells expressing mutant FLAG-H2A K118-119R, band 2 was induced upon expression of BC-R/BD-R in these cells. Band 2 was not observed in cells expressing FLAG-H2A K124-127-129R mutant, suggesting that ubiquitylation required one or more of the three most C-terminal lysine residues of H2A. We observed a similar requirement for K125, K127, and K129 for ubiquitylation of FLAG-H2A K118-119R in HEK293 cells expressing the BDfBC RING domain complex (Figure S3D). Although these data highlight the importance of K127 for ubiquitylation of H2A, they did not establish which of the lysine residues (K125, K127, and K129) becomes conjugated to ubiquitin.

Given that H2Aub comprises only 5%–10% of all H2A in cells, the amount of H2Aub that was modified at lysine residues other than K118-119 was extremely low (Figures 3D and 3F). Expression of the RING fusion protein BDfBC in HEK293 cells significantly increased the cellular pool of H2Aub, indicating that H2A is an efficient substrate for this E3 even when it is not specifically directed to chromatin as a fusion protein with LacI (Figure 3F). By contrast, we observed no increase in H2Bub after expression of BDfBC (Figure S2B). Next, we purified chromatin-associated H2Aub from cells expressing BDfBC and analyzed it by mass spectrometry. The number and close proximity of lysine

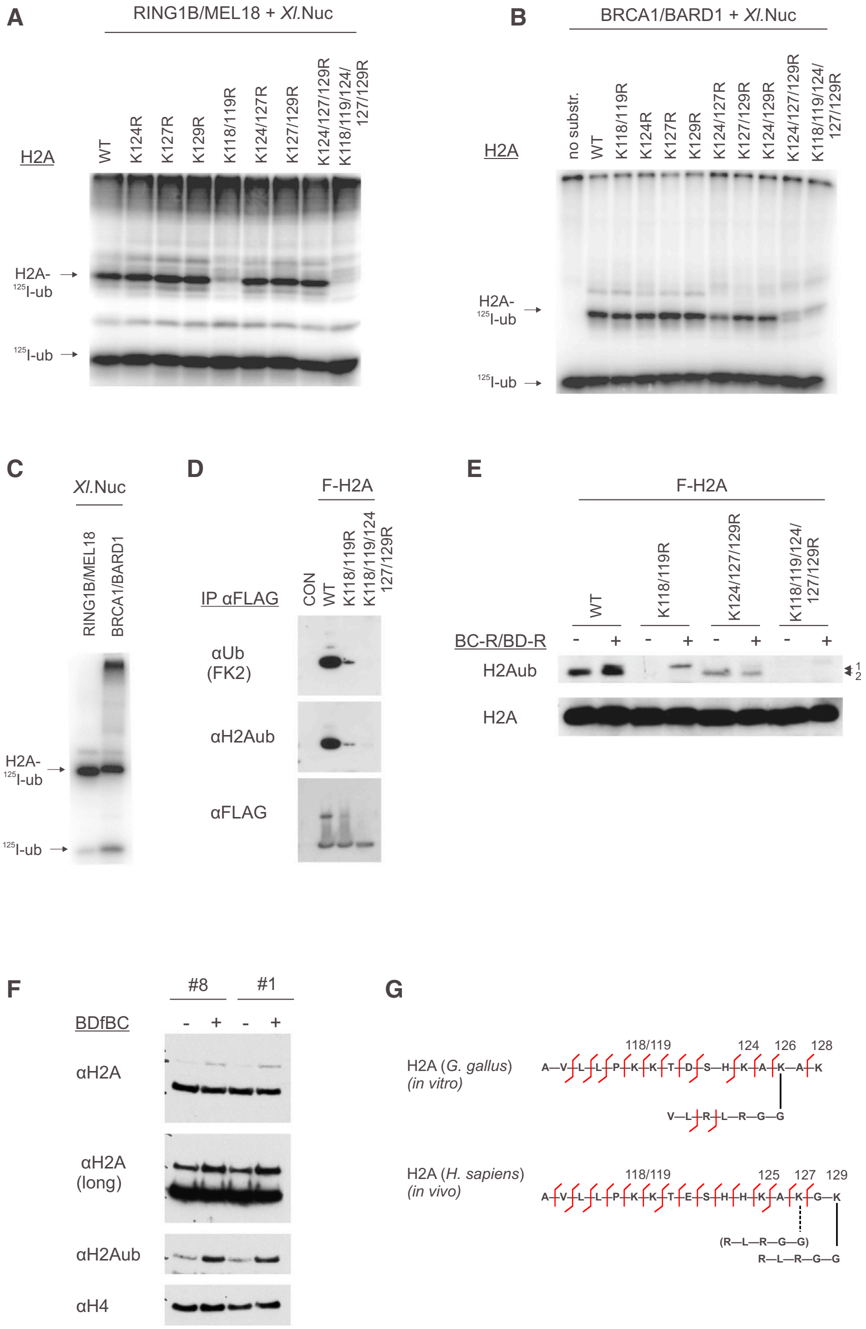


**Figure 3. BRCA1/BARD1 Ubiquitylates K127-129 of Histone H2A In Vitro and In Vivo**

(A) Ubiquitylation of nucleosomes by RING1B/MEL18 is impaired by mutation of lysines 118 and 119 of histone H2A.

(B) Ubiquitylation of nucleosomes by BRCA1/BARD1 is reduced for histone H2A mutated at lysines K124, K127, and K129. Small amounts of diubiquitylated histone were observed as indicated (*).

(C) Ubiquitylation of recombinant *Xenopus* nucleosomes by BRCA1/BARD1 and RING1B/MEL18, showing the difference in migration of the H2Aub product.

(D) FLAG-H2A was stably expressed in DT40 cells and immunoprecipitated under denaturing conditions as described previously (Wang et al., 2004). Cells expressing wild-type or mutant H2A are indicated. Immunoprecipitated proteins were analyzed by western blot and probed with antibodies against FLAG, ubiquitin (FK2), and ubiquityl-H2A (E6C5).

(E) DT40 cell lines expressing wild-type or mutant FLAG-H2A (K118/119R, K124/127/129, and K118/119/124/127/129) were transfected with the RING domains of BRCA1 and BARD1. Cells were harvested after 48 hr and histone proteins were isolated by acid extraction. FLAG-H2Aub was detected by western blot with anti-FLAG after separation by PAGE. H2Aub was detected after expression of the BRCA1 and BARD1 RING domains (band 1) and/or by endogenous E3 activity (band 2).

(F) HEK293 cells expressing BDfBC-EGFP-NLS protein under the control of a Tet-responsive promoter was induced overnight with 1 μg/ml doxycycline. Nuclei were prepared from the cells and acid-extracted histones were separated on a 12% BisTris Novex gel and probed with the indicated antibodies. Increased H2Aub dependent on expression of BDfBC is indicated for two independent clones (#8 and #1).

(G) Top, BRCA1/BARD1 ubiquitylates chicken histone H2A at lysine 126 (equivalent to K127 in humans) in nucleosomes. The modified peptide fragments identified by mass spectrometry are indicated. Data supporting this modification are provided in Figure S3E. Bottom, BDfBC expressed in HEK293 cells ubiquitylates human histone H2A at lysine K127-129. H2Aub was recovered from chromatin as described in the Experimental Procedures. The modified peptide fragments identified by mass spectrometry are indicated in the illustration. Data supporting this modification are provided in Figure S3F.

residues required us to digest H2Aub with pepsin at pH 1.3 rather than trypsin prior to mass spectrometry analysis. We found that although the majority of cellular H2Aub was modified on K118 or K119, expression of BDfBC coincided with the recovery of ubiquitylated peptide corresponding to the C-terminal residues GK (residues 128-129) of H2A (Figures 3G, bottom, and S3F). We also identified peptides consistent with low-level ubiquitin conjugation at K127 (Figure S3G). On the basis of these data, the primary acceptor for BRCA1 E3 activity is probably lysine 129 of histone H2A. However, lysine 127, which is important for efficient ubiquitylation of H2A by BRCA1/BARD1 in vitro and in vivo, may also be ubiquitylated.

## DISCUSSION

To date, neither the substrate nor the function of the BRCA1/BARD1 ubiquitin ligase has been well established. We have established that BRCA1/BARD1 specifically ubiquitylates histone H2A in chromatin in vitro and in vivo. Our data support a role for BRCA1/BARD1 as a histone-H2A-specific ubiquitin ligase

that ubiquitylates the C-terminal tail of H2A at the previously uncharacterized lysine residues K127-129.

Several pieces of evidence link BRCA1-dependent E3 activity with ubiquitylation of histones in chromatin. First, purified BRCA1/BARD1 ubiquitylates individual histone proteins in vitro, albeit with little specificity (Chen et al., 2002; Hashizume et al., 2001; Mallery et al., 2002). We show that the specificity of BRCA1/BARD1 for K127-129 of H2A is acquired only in a nucleosomal context, a characteristic that is shared with RING1B/MEL18 for ubiquitylation of H2Aub118-119 (Elderkin et al., 2007). Moreover, our data suggest that the ability to discriminate nucleosome substrate from free histone in vivo resides within the heterodimeric RING domains comprising amino acids 1–109 of BRCA1 and 1–126 of BARD1.

Second, the RING domains of BRCA1/BARD1 that confer its E3 activity are structurally related to the RING1B/MEL18 RING domain subunits of the histone-H2A-specific ubiquitin ligase PRC1 (Brzovic et al., 2006; Buchwald et al., 2006; Li et al., 2006). Of note, the basic patches on the surface of RING1B/BMI1, which have been shown to be involved in DNA binding of the E3-UbcH5 complex on nucleosomes (Bentley et al., 2011), are conserved in BRCA1, but not in BARD1. However, it is unclear whether this might affect the position of E3 relative to its nucleosome substrate.

We note that BRCA1/BARD1 and RING1B/MEL18 are heterodimeric E3 ligases (Brzovic et al., 2001; Buchwald et al., 2006; Li et al., 2006), whereas RNF168 is monomeric (Campbell et al., 2012) and therefore might interact with its chromatin substrate in a different manner (Mattiroli et al., 2012). Recent evidence suggests that ubiquitylation of histone H2A by RING1B/BMI1 and RNF168 is dependent on an acidic patch present on the exposed surface of nucleosomes. Expression of a peptide that interfered with binding to this acidic patch caused a reduction in DNA-damage-induced H2Aub by RNF168 and a concomitant failure to recruit BRCA1 at sites of DNA damage in vivo (Leung et al., 2014; Mattiroli et al., 2014). However, recruitment of BRCA1 at sites of DNA damage is dependent on RNF168-mediated ubiquitylation, and therefore no conclusion can be made regarding the effect on BRCA1 E3 ligase activity.

Third, our data support recent evidence indicating that small interfering RNA (siRNA)-mediated knockdown of BRCA1 results in derepression of satellite DNA with an accompanying loss of H2Aub in this region (Zhu et al., 2011). Our findings highlight the potential for BRCA1 to function directly in this process through ubiquitylation of histone H2A. This is consistent with the demonstration by Zhu et al. (2011) that repression of satellite DNA can be restored by expression of histone H2A protein fused at its C terminus with ubiquitin. Moreover, it suggests that although ubiquitylation of H2A on K127-129 is characteristic of BRCA1/BARD1 E3 function, the exact position of this ubiquitin in the C-terminal tail might not be critical for its role in repression of satellite DNA. It is possible that H2AubK127-129 and the more common H2Aub119 perform very similar functions in chromatin, and that BRCA1/BARD1 targets this modification to specific regions of chromatin.

BRCA1 has been linked to ubiquitin conjugates on chromatin at sites of DNA breaks. However, previous attempts to visualize the ubiquitylated products of BRCA1/BARD1 E3 activity at sites of DNA damage have been hampered by its complex recruitment to sites of DNA breaks involving a ubiquitin-mediated signaling pathway and several different ubiquitin ligases (Gospodinov and Herceg, 2013). Here, we show that site-specific targeting of the BRCA1/BARD1 RING domains is sufficient for local ubiquitylation of H2A in vivo. Mass spectrometry analysis confirmed that the biochemical activity and specificity of the BDfBC complex to ubiquitylate the C-terminal tail of H2A on lysines K127-129 in vivo reflect those of the full-length BRCA1/BARD1 complex in vitro. Moreover, using the "E3-dead" I26A mutant protein, we established that generation of H2Aub by BDfBC is specifically dependent on BRCA1 E3 activity.

Previous studies led us to believe that the position of ubiquitin within a nucleosome is more important for its function than the exact location of the modification within the histone tail. Indeed, it has been shown that ubiquitin fused to the very C-terminal amino acid of histone H2A contributes to the repression of satellite DNA. The exact role(s) of ubiquitin, however, is still enigmatic.

The contribution of BRCA1 E3 activity to the DNA damage response is unclear. Mice expressing the enzymatically dead mutant BRCA1(I26A) are no more tumor prone than those expressing wild-type protein (Shakya et al., 2011). On the other hand, murine embryonic stem cells expressing this BRCA1 I26A mutant protein have increased levels of genomic aberrations (Reid et al., 2008). It is possible that rather than promoting repair, the BRCA1 ubiquitin ligase functions as a negative regulator of double-strand break repair, as was shown recently for the ubiquitin-binding RAP80 complex (Coleman and Greenberg, 2011).

Although the role of monoubiquitylated H2A at DNA breaks is still unclear, our data establish the potential of BRCA1/BARD1 as a specific H2A ubiquitin ligase on nucleosome substrates. Moreover, the identification of a previously uncharacterized form of H2Aub that might be uniquely associated with the E3 activity of BRCA1 raises the possibility of generating antibodies directed against H2AubK127-129 for use as a diagnostic tool to identify biochemically active BRCA1.

## EXPERIMENTAL PROCEDURES

### Cell Culture

DT40 chicken cells were propagated in standard media supplemented with RPMI (Invitrogen) at 37°C, 6% $CO_2$. HeLa cells were cultured in Dulbecco's modified Eagle's medium (DMEM; Invitrogen) supplemented with 10% fetal calf serum. HEK293 FlpIn T-Rex cells (Invitrogen) were grown in DMEM containing 10% tetracycline-free fetal bovine serum.

### Microscopy

Cells were prepared as described in the Supplemental Experimental Procedures and visualized using an LSM510 confocal microscope (Leica).

### Ubiquitylation of Recombinant Nucleosomes and Chromatin

Recombinant *X. laevis* histones were expressed and purified from *E. coli* and reconstituted into nucleosomes as described in the Supplemental Experimental Procedures. The chicken histone octamers were a kind gift from Professor Daniela Rhodes and were purified from chicken erythrocyte nuclei as described previously (Thomas and Butler, 1980). For nucleosome ubiquitylation, chromatin or individual histones were incubated with 200 ng E1 (affinity or Boston Biochem), 200 ng UbcH5c (affinity or Boston Biochem), 1 μg

ubiquitin (Sigma), 1 mM ATP and 0.1 μg purified E3 were in a reaction volume of 10 μl for 15 min, followed by addition of 10 μl of 0.5 μg $^{125}$I-ubiquitin, substrate and 1 mM in 1x ub buffer (Mallery et al., 2002). Reactions were stopped by addition of SDS buffer and applied for gel electrophoresis.

### Purification of Ubiquitylated Histones and Mass Spectrometry

H2Aub was isolated from cells by initial purification of H2A and H2B from chromatin in cells using the Histone Purification Kit (Active Motif). Histones were separated by PAGE using 12% Bis-Tris gel (Invitrogen) and MES buffer, and Coomassie-stained H2Aub bands were excised. In-gel digestion with pepsin (pH 1.3) was performed and peptides were analyzed by nanoLC-LTQ/Orbitrap in a data-dependent tandem mass spectrometry mode. For nucleosomes ubiquitylated in vitro, in-gel digestion was also performed with pepsin and analyzed by NextGen Bioscience.

## SUPPLEMENTAL INFORMATION

Supplemental Information includes Supplemental Experimental Procedures and three figures and can be found with this article online at http://dx.doi.org/10.1016/j.celrep.2014.07.025.

## AUTHOR CONTRIBUTIONS

R.K., D.L.M., C.L., and K.H. conceived and designed experiments. R.K., D.L.M., and C.L. performed experiments. J.T.J.H. performed mass spectrometry and analyzed the results. R.K. and K.H. wrote the manuscript.

## ACKNOWLEDGMENTS

We thank Professor Daniela Rhodes, Dr. Andrew Routh, and Dr. Sara Sandin for the kind gift of purified chicken histone octamers and for their helpful advice. We would also like to thank Dr. Susan Janicki (Wistar Institute) for U2OS cell line 2-6-3, Professor Rachel Klevit (University of Washington) for pBD126FBC109, and Dr. Roger Greenberg (University of Pennsylvania) for pSVFokI-mCherryLacI. We thank Dr. Sarah Elderkin (Babraham Institute) for histone-expressing plasmids and advice, and Dr. Patrick Varga-Weiss (Babraham Institute) for technical advice. We are grateful to Dr. Richard Jones (NextGen Bioscience) for mass spectrometry analysis of in vitro modified chromatin. This work was supported by the Medical Research Council (U105184300 to R.K. and K.H.), an MRC-LMB International Postdoctoral Fellowship to R.K., a short-term fellowship from the Max Perutz Fund (Karn Donation), funds from the Ninewells Cancer Campaign to C.L., and Cancer Research UK (C13906/A12769 to K.H.).

Received: November 25, 2013
Revised: March 18, 2014
Accepted: July 16, 2014
Published: August 14, 2014

## REFERENCES

Bentley, M.L., Corn, J.E., Dong, K.C., Phung, Q., Cheung, T.K., and Cochran, A.G. (2011). Recognition of UbcH5c and the nucleosome by the Bmi1/Ring1b ubiquitin ligase complex. EMBO J. *30*, 3285–3297.

Brzovic, P.S., Rajagopal, P., Hoyt, D.W., King, M.C., and Klevit, R.E. (2001). Structure of a BRCA1-BARD1 heterodimeric RING-RING complex. Nat. Struct. Biol. *8*, 833–837.

Brzovic, P.S., Lissounov, A., Christensen, D.E., Hoyt, D.W., and Klevit, R.E. (2006). A UbcH5/ubiquitin noncovalent complex is required for processive BRCA1-directed ubiquitination. Mol. Cell *21*, 873–880.

Buchwald, G., van der Stoop, P., Weichenrieder, O., Perrakis, A., van Lohuizen, M., and Sixma, T.K. (2006). Structure and E3-ligase activity of the Ring-Ring complex of polycomb proteins Bmi1 and Ring1b. EMBO J. *25*, 2465–2474.

Campbell, S.J., Edwards, R.A., Leung, C.C., Neculai, D., Hodge, C.D., Dhe-Paganon, S., and Glover, J.N. (2012). Molecular insights into the function of RING finger (RNF)-containing proteins hRNF8 and hRNF168 in Ubc13/Mms2-dependent ubiquitylation. J. Biol. Chem. *287*, 23900–23910.

Chen, A., Kleiman, F.E., Manley, J.L., Ouchi, T., and Pan, Z.Q. (2002). Autoubiquitination of the BRCA1*BARD1 RING ubiquitin ligase. J. Biol. Chem. *277*, 22085–22092.

Christensen, D.E., Brzovic, P.S., and Klevit, R.E. (2007). E2-BRCA1 RING interactions dictate synthesis of mono- or specific polyubiquitin chain linkages. Nat. Struct. Mol. Biol. *14*, 941–948.

Coleman, K.A., and Greenberg, R.A. (2011). The BRCA1-RAP80 complex regulates DNA repair mechanism utilization by restricting end resection. J. Biol. Chem. *286*, 13669–13680.

Doil, C., Mailand, N., Bekker-Jensen, S., Menard, P., Larsen, D.H., Pepperkok, R., Ellenberg, J., Panier, S., Durocher, D., Bartek, J., et al. (2009). RNF168 binds and amplifies ubiquitin conjugates on damaged chromosomes to allow accumulation of repair proteins. Cell *136*, 435–446.

Elderkin, S., Maertens, G.N., Endoh, M., Mallery, D.L., Morrice, N., Koseki, H., Peters, G., Brockdorff, N., and Hiom, K. (2007). A phosphorylated form of Mel-18 targets the Ring1B histone H2A ubiquitin ligase to chromatin. Mol. Cell *28*, 107–120.

Gospodinov, A., and Herceg, Z. (2013). Shaping chromatin for repair. Mutat. Res. *752*, 45–60.

Hashizume, R., Fukuda, M., Maeda, I., Nishikawa, H., Oyake, D., Yabuki, Y., Ogata, H., and Ohta, T. (2001). The RING heterodimer BRCA1-BARD1 is a ubiquitin ligase inactivated by a breast cancer-derived mutation. J. Biol. Chem. *276*, 14537–14540.

Leung, J.W., Agarwal, P., Canny, M.D., Gong, F., Robison, A.D., Finkelstein, I.J., Durocher, D., and Miller, K.M. (2014). Nucleosome acidic patch promotes RNF168- and RING1B/BMI1-dependent H2AX and H2A ubiquitination and DNA damage signaling. PLoS Genet. *10*, e1004178.

Li, Z., Cao, R., Wang, M., Myers, M.P., Zhang, Y., and Xu, R.M. (2006). Structure of a Bmi-1-Ring1B polycomb group ubiquitin ligase complex. J. Biol. Chem. *281*, 20643–20649.

Mallery, D.L., Vandenberg, C.J., and Hiom, K. (2002). Activation of the E3 ligase function of the BRCA1/BARD1 complex by polyubiquitin chains. EMBO J. *21*, 6755–6762.

Mattiroli, F., Vissers, J.H., van Dijk, W.J., Ikpa, P., Citterio, E., Vermeulen, W., Marteijn, J.A., and Sixma, T.K. (2012). RNF168 ubiquitinates K13-15 on H2A/H2AX to drive DNA damage signaling. Cell *150*, 1182–1195.

Mattiroli, F., Uckelmann, M., Sahtoe, D.D., van Dijk, W.J., and Sixma, T.K. (2014). The nucleosome acidic patch plays a critical role in RNF168-dependent ubiquitination of histone H2A. Nat. Commun. *5*, 3291.

Morris, J.R., and Solomon, E. (2004). BRCA1 : BARD1 induces the formation of conjugated ubiquitin structures, dependent on K6 of ubiquitin, in cells during DNA replication and repair. Hum. Mol. Genet. *13*, 807–817.

Moynahan, M.E., and Jasin, M. (2010). Mitotic homologous recombination maintains genomic stability and suppresses tumorigenesis. Nat. Rev. Mol. Cell Biol. *11*, 196–207.

Paull, T.T., Cortez, D., Bowers, B., Elledge, S.J., and Gellert, M. (2001). Direct DNA binding by Brca1. Proc. Natl. Acad. Sci. USA *98*, 6086–6091.

Reid, L.J., Shakya, R., Modi, A.P., Lokshin, M., Cheng, J.T., Jasin, M., Baer, R., and Ludwig, T. (2008). E3 ligase activity of BRCA1 is not essential for mammalian cell viability or homology-directed repair of double-strand DNA breaks. Proc. Natl. Acad. Sci. USA *105*, 20876–20881.

Ruffner, H., Joazeiro, C.A., Hemmati, D., Hunter, T., and Verma, I.M. (2001). Cancer-predisposing mutations within the RING domain of BRCA1: loss of ubiquitin protein ligase activity and protection from radiation hypersensitivity. Proc. Natl. Acad. Sci. USA *98*, 5134–5139.

Shakya, R., Reid, L.J., Reczek, C.R., Cole, F., Egli, D., Lin, C.S., deRooij, D.G., Hirsch, S., Ravi, K., Hicks, J.B., et al. (2011). BRCA1 tumor suppression depends on BRCT phosphoprotein binding, but not its E3 ligase activity. Science *334*, 525–528.

Stewart, G.S., Panier, S., Townsend, K., Al-Hakim, A.K., Kolas, N.K., Miller, E.S., Nakada, S., Ylanko, J., Olivarius, S., Mendez, M., et al. (2009). The

RIDDLE syndrome protein mediates a ubiquitin-dependent signaling cascade at sites of DNA damage. Cell *136*, 420–434.

Thomas, J.O., and Butler, P.J. (1980). Size-dependence of a stable higher-order structure of chromatin. J. Mol. Biol. *144*, 89–93.

Venkitaraman, A.R. (2001). Functions of BRCA1 and BRCA2 in the biological response to DNA damage. J. Cell Sci. *114*, 3591–3598.

Wang, H., Wang, L., Erdjument-Bromage, H., Vidal, M., Tempst, P., Jones, R.S., and Zhang, Y. (2004). Role of histone H2A ubiquitination in Polycomb silencing. Nature *431*, 873–878.

Wu, L.C., Wang, Z.W., Tsan, J.T., Spillman, M.A., Phung, A., Xu, X.L., Yang, M.C., Hwang, L.Y., Bowcock, A.M., and Baer, R. (1996). Identification of a RING protein that can interact in vivo with the BRCA1 gene product. Nat. Genet. *14*, 430–440.

Xu, B., Kim St, and Kastan, M.B. (2001). Involvement of Brca1 in S-phase and G(2)-phase checkpoints after ionizing irradiation. Mol. Cell. Biol. *21*, 3445–3450.

Zhu, Q., Pao, G.M., Huynh, A.M., Suh, H., Tonnu, N., Nederlof, P.M., Gage, F.H., and Verma, I.M. (2011). BRCA1 tumour suppression occurs via heterochromatin-mediated silencing. Nature *477*, 179–184.

**Cell Reports, Volume *8***

**Supplemental Information**

# BRCA1 Is a Histone-H2A-Specific Ubiquitin Ligase

**Reinhard Kalb, Donna L. Mallery, Conor Larkin, Jeffrey T.J. Huang, and Kevin Hiom**

**Experimental Procedures**

**Cell culture**

DT40 chicken cells were propagated in standard RPMI (Invitrogen) supplemented media at 37 °C, 6% $CO_2$. Transfections were carried out by electroporation as previously described (Mallery et al., 2002). Hela cells were cultured in DMEM (Invitrogen) supplemented with 10% FCS. HEK293 FlpIn T-Rex cells (Invitrogen) were grown in DMEM containing 10% tetracycline free FBS and 50 U/ml Penicillin, 50 μg/ml Streptomycin at 37˚C in 5% $CO_2$. BDfBC EGFP-NLS expressing cell lines were generated according to the manufacturer instructions. Individual clones were selected with 200 μg/ml Hygromycin B (Invitrogen). 2-6-3 cells containing the LacO array were cultured in DMEM supplemeneted with 10% FCS, 50U/ml Penicillin, 50 μg/ml Streptomycin, 100 μg/ml Hygromycin B at 37˚C in 5% $CO_2$.

**BDfBC construct**

The Bard1-Brca1 RING fusion construct was a gift from Rachel Klevit. The ORF was amplified and cloned into pSV2-Fok1 mCherryLacI (a gift from Roger Greenberg) digested with NheI and AgeI. Bard1-Brca1 RING – EGFP-NLS fusion construct was cloned by opening pSV2-BaBr mCherryLacI with AgeI and Kpn2I ligation of PCR amplified EGFP containing the SV40 NLS sequence to the C-terminus of EGFP to create BDfBC EGFP-NLS. BDfBC EGFP-NLS was then subcloned into the KpnI and NotI sites of pcDNA5/FRT/TO. I26A mutants of the Brca1 RING fusion were generated with the Quickchange Lightening Site directed mutagenesis kit. Flag-H2A

mutants were expressed from the pDest12.2 expression vector under the control of the CMV promoter. C-terminal tail mutants were generated by amplification of the H2A coding sequence using a reverse primer containing the relevant mutation(s). Sequences of the mutagenic primers are available on request. The mutant cDNA was then re-cloned back into pDest12.2 using standard molecular biology techniques. All plasmids were verified by sequencing.

**Microscopy**

2-6-3 cells were seeded at a density of 2.5 x $10^4$ cells/$cm^2$ in a 12 well dish containing a coverslip. 24 hours later the cells were transfected with the relevant mCherryLacI constructs using XtermeGENE HP (Roche) according to manufacturers instructions. After 24 h cells were washed with PBS and then incubated with CKS buffer (10 mM PIPES pH7.0, 100 mM NaCl, 300 mM Sucrose, 3 mM $MgCl_2$) supplemented with 0.5% Triton-X-100 and protease inhibitors for 3 minutes at room temperature. Washed cells were fixed with 4% Paraformaldehyde for 10 minutes at room temperature, permeabilised in 0.5% Triton-X-100 in PBS for 3 x 5 minutes and blocked for 30 minutes at 37˚C in blocking buffer (1% BSA in PBS). Fixed cells were incubated with primary antibody at the relevant dilution in blocking buffer for 1 hour at 37˚C, washed with PBS and incubated with the appropriate secondary antibody, 250 μg/ml RNase A (Qiagen) and 1μM TO-PRO-3 for 30 minutes. After another wash in the dark the coverslips were mounted onto slides using ProLong GOLD (Invitrogen). Antibodies used were anti-H2Aub (Clone E6C5) (Millipore) 1:100 and FK2 (Enzo Life Science) 1:500. Appropriate AlexaFluor 488 secondary

antibodies (Invitrogen) were used at 1:1000. Images were captured on a Zeiss LSM510 confocal microscope using the 40x oil immersion objective (N/A 1.3). At least 100 mCherry foci were counted for each condition.

**Proteins**

C-terminal tagged FLAG/12xHis (FH) full-length human tagged BRCA1 and BARD1 cDNA was cloned into pDEST8 and expressed in Sf9 using the Bac-to-Bac baculovirus expression system (Invitrogen) and purified essentially as described in Mallery et al. (2002). In short, viruses with high expression levels for BRCA1-FH and BARD1-FH were co-infected in Sf9 insect cells for 48 h. Cells were harvested by centrifugation at 1200 rpm for 5 min. Cleared cell lysates were prepared from fresh cells by sonication in nickel lysis buffer (20 mM Tris–HCl pH 7.5, 500 mM KCl, 10% glycerol, 1% Triton X-100, 2 mM 2-mercapto ethanol) containing 5 mM imidazole and complete protease inhibitors (Roche), followed by ultracentrifugation at 35 000 rpm for 45 min. The lysate was loaded directly onto a 5 ml HiTrap $Ni^{2+}$ chelating column (GE Healthcare) and washed with 10 column volumes of buffer A (20 mM Tris–HCl pH 7.5, 500 mM KCl, 10% glycerol, 0.1% Triton X-100, 2 mM 2-mercapto ethanol) followed by at least 10 column volumes of buffer A containing 60 mM imidazole. Proteins were eluted in 10 ml of buffer A containing 750 mM imidazole, and diluted 3.3 fold in HepA buffer (20 mM Tris-HCl (pH 7.4), 10% glycerol, 0.1% Triton-X 2 mM DTT) to give a final concentration of 150 mM KCl. The $Ni^{2+}$ eluate was loaded onto a 1 ml heparin column (GE Healthcare) and washed with at least 10 cloumn columns HepA containing 150 mM KCl. BRCA1/BARD1 was eluted with a 150 mM -1 M KCl gradient. Peak protein

fractions, which eluted at approx. 800 mM KCl, were dialysed against storage buffer (25 mM Tris–HCl pH 7.5, 150 mM KCl, 10% glycerol and 2 mM DTT). Aliquots were frozen on liquid nitrogen and stored at -80°C.

BRCA1(I26A)-FH/BARD1-HA, Ring1B-FH/Mel18-HA, BC-R/BD-R (both FLAG, 12xHis tagged) were expressed as described for BRCA1/BARD1 and in (Elderkin et al., 2007). Purification was performed by $Ni^{2+}$ affinity chromatography (Ni-NTA, Qiagen).

**Reconstitution of recombinant *Xenopus laevis* nucleosomes**

Histones (H2A, H2B, H3 and H4) were expressed in BL21(DE3)LysS and purified essentially as described by Luger and coworkers (Luger, 1996). Bacteria were lysed in 50 mM Tris-HCl pH7.4, 100 mM NaCl, 1 mM EDTA and proteinase inhibitors. Inclusion bodies were pelleted by centrifugation, washed several times w/o 1% Triton-X100 and solved in unfolding buffer (20 mM Tris-HCl pH7.4, 7 M guanidium HCl, 10 mM DTT). Equimolar amounts of core histones were mixed and dialysed against refold buffer (2M NaCl, 10 mM Tris-HCl pH 7.4, 5 mM 2-mercaptoethanol). Histone octamers were separated by gel filtration (Superdex S200, GE Healthcare). Equal amounts of octamers (recombinant *X. laevis* or purified from chicken erythrocyte chromatin) and 5S rDNA were used for nucleosome reconstitution via salt dialysis. Reconstituted nucleosomes were stored in TE buffer at 4 °C.

**Nucleosome Aassembly**

Nucleosomes were assembled using purified octamers (recombinant *X. laevis* or chicken) and polyglutamic acid (PGA; Sigma P4886) according to Stein et

al. (Stein et al., 1979). PGA and histones were mixed in a 2:1 ratio in 150 mM NaCl and incubated for 1-2 h at room temperature. Precipitates were removed by centrifugation and the supernatant (HP-mix) was stored at 4 °C. Different ratios of $^{32}P$-body labeled DNA (Widom 601 sequence, 161 nt) and HP-mix were empirically tested to reveal the optimal conditions for nucleosome assembly. Nucleosomes were separated by native polyacrylamide gel electrophoresis (4.5% polyacrylamide, 0.25x TBE, 5% glycerol), assembled nucleosomes were cut out and eluted from the gel.

**Binding assay**

Approx. 10-40 fmol of gel eluted nucleosomes were incubated for 10 min on ice with BRCA1/BARD1 full-length proteins. The sample was then loaded on a native polyacrylamide gel (4.5% polyacrylamide, 0.4x TBE, 5% glycerol) and separated by gel electrophoresis. Migration of radiolabelled nucleosomes were detected by autoradiography of the dried gel (Phosphoimager, GE Healthcare).

**Ubiquitylation Assays**

The radioactive ubiquitylation assay was done as described previously (Mallery et al., 2002) with minor modifications. 200 ng E1 (affinity or Boston Biochem), 200 ng UbcH5c (affinity or Boston Biochem), 1 µg ubiquitin (Sigma), 1 mM ATP and 0.1 µg purified E3 were incubated in a reaction volume of 10 µl for 15 min, followed by additon of 10 µl of 0.5 µg $^{125}I$-ubiquitin, substrate and 1 mM in 1x ub buffer. Reaction were stopped by addition of SDS buffer and applied for gel electrophoresis. $^{125}I$-labeled products were

visualized using the Molecular Dynamics Typhoon Phosphorimager (GE Healthcare) and ImageQuaNT software. Non radioactive ubiquitylation was performed in a 20 µl reaction containing 50 ng E1, 50 ng UbcH5c, 0.5 µg ubiquitin and 0.8-1.0 µg 61*197 NRL nucleosome arrays/ native chromatin with 50 ng E3. Reaction was stopped by addition of SDS loading buffer.

**Immunochemistry**

For *in vivo* experiments chicken DT40 cell lines stably expressing FLAG-H2A (WT, K119R/K120R, K125R/K128R/K130R and K119R/K120R/K125R/K128R/K130R) were established. FLAG-H2A was bound to anti-FLAG M2 resign under denaturing conditons (Sigma) as described previously (Doil et al., 2009; Shiio and Eisenman, 2003). After elution with FLAG peptide the sample was separated via SDS PAGE and used for immunodetection. Pull down was checked by Coomassie staining. The ubiquitin E3 ligase activity of BRCA1/BARD1 on H2A was tested by transient co-expression of BRCA1 and BARD1 RING domains in indicated cell lines. 48h after transfection histones were isolated by acid extraction and immunoblotted using anti-FLAG antibody (M2, Sigma).

Histones of BDfBC cell lines were acid extracted. In short, cells were seeded at a density of 6 x 104 cells/cm$^2$ in a 10 cm dish. 24 h after transfection with plasmids carrying the ORF of Flag-H2A mutants the expression of BDfBC EGFP-NLS was induced with 5 µg/ml Dox. Cells were harvested 16-24 hours after induction, washed once with PBS and then resuspended in Buffer A (10 mM Hepes pH 7.0, 10mM KCl, 1.5Mm $MgCl_2$, 340 mM Sucrose, 10% Glycerol, 10mM NEM, 0.02% $NaN_3$ and 1 x Halt Protease Inhibitors (Thermo

Scientific) supplemented with 0.1% Triton-X-100). The samples were incubated on ice for 8 minutes then centrifuged at 1,300 x g for 5 minutes at 4˚C, washed once with Buffer A and then lysed in Buffer B (3mM EDTA, 0.2mM EGTA, 10 mM NEM, 0.02% $NaN_3$ and 1 x Halt Protease Inhibitors (Thermo Scientific)) on ice to release the chromatin from the nuclei. Samples were centrifuged at 1,700 x g for 5 minutes at 4˚C to separate the chromatin from the soluble nuclear fraction. The soluble nuclear fraction was retained to verify expression of the BDfBC EGFP-NLS. The resulting pellet was washed for 10 minutes with Buffer B supplemented with 350mM NaCl to remove loosely bound chromatin proteins. Following centrifugation at 1,700 x g for 5 minutes at 4˚C, 200 mM HCl was added to the resulting pellet and incubated overnight at 4˚C to extract the histones. Extracted histones were neutralized by addition of 1/5th the final volume of 2.5M Tris-Cl pH 8.0. The protein concentration was estimated using a Bradford assay. Equal amounts of protein were resolved on a 12% Bis-Tris gel ran using MES buffer. The proteins were transferred to a nitrocellulose membrane and probed with the appropriate antibodies. Antibodies used were Anti-Flag M2 (Sigma) 1:1000, Anti-GFP Clone B2 (Santa Cruz) 1:1000, Anti-H2B N-20 (Santa Cruz) 1:1000 and Anti-p84 clone 5E10 (GeneTex) 1:2000.

**Mass spectrometry**

H2Aub was purified from cells using Actif motif histone purification kit followed by gel purification of H2Aub after PAGE in a 12% Bis-Tris acrylamide gel using MES buffer. H2Aub was digested in gel and analyzed as described in Figure S3.

**References**

Brzovic, P.S., Rajagopal, P., Hoyt, D.W., King, M.C., and Klevit, R.E. (2001). Structure of a BRCA1-BARD1 heterodimeric RING-RING complex. Nature structural biology *8*, 833-837.

Buchwald, G., van der Stoop, P., Weichenrieder, O., Perrakis, A., van Lohuizen, M., and Sixma, T.K. (2006). Structure and E3-ligase activity of the Ring-Ring complex of polycomb proteins Bmi1 and Ring1b. EMBO J *25*, 2465-2474.

Doil, C., Mailand, N., Bekker-Jensen, S., Menard, P., Larsen, D.H., Pepperkok, R., Ellenberg, J., Panier, S., Durocher, D., Bartek, J*., et al.* (2009). RNF168 binds and amplifies ubiquitin conjugates on damaged chromosomes to allow accumulation of repair proteins. Cell *136*, 435-446.

Elderkin, S., Maertens, G.N., Endoh, M., Mallery, D.L., Morrice, N., Koseki, H., Peters, G., Brockdorff, N., and Hiom, K. (2007). A phosphorylated form of Mel-18 targets the Ring1B histone H2A ubiquitin ligase to chromatin. Mol Cell *28*, 107-120.

Huynh, V.A., Robinson, P.J., and Rhodes, D. (2005). A method for the in vitro reconstitution of a defined "30 nm" chromatin fibre containing stoichiometric amounts of the linker histone. J Mol Biol *345*, 957-968.

Lowary, P.T., and Widom, J. (1998). New DNA sequence rules for high affinity binding to histone octamer and sequence-directed nucleosome positioning. J Mol Biol *276*, 19-42.

Luger, K. (2003). Structure and dynamic behavior of nucleosomes. Curr Opin Genet Dev *13*, 127-135.

Mallery, D.L., Vandenberg, C.J., and Hiom, K. (2002). Activation of the E3 ligase function of the BRCA1/BARD1 complex by polyubiquitin chains. EMBO J *21*, 6755-6762.

Robinson, P.J., Fairall, L., Huynh, V.A., and Rhodes, D. (2006). EM measurements define the dimensions of the "30-nm" chromatin fiber: evidence for a compact, interdigitated structure. Proc Natl Acad Sci U S A *103*, 6506-6511.

Routh, A., Sandin, S., and Rhodes, D. (2008). Nucleosome repeat length and linker histone stoichiometry determine chromatin fiber structure. Proc Natl Acad Sci U S A *105*, 8872-8877.

Shiio, Y., and Eisenman, R.N. (2003). Histone sumoylation is associated with transcriptional repression. Proc Natl Acad Sci U S A *100*, 13225-13230.

Stein, A., Whitlock, J.P., Jr., and Bina, M. (1979). Acidic polypeptides can assemble both histones and chromatin in vitro at physiological ionic strength. Proc Natl Acad Sci U S A *76*, 5000-5004.

Thomas, J.O., and Butler, P.J. (1980). Size-dependence of a stable higher-order structure of chromatin. J Mol Biol *144*, 89-93.

## Supplemental figures and legends

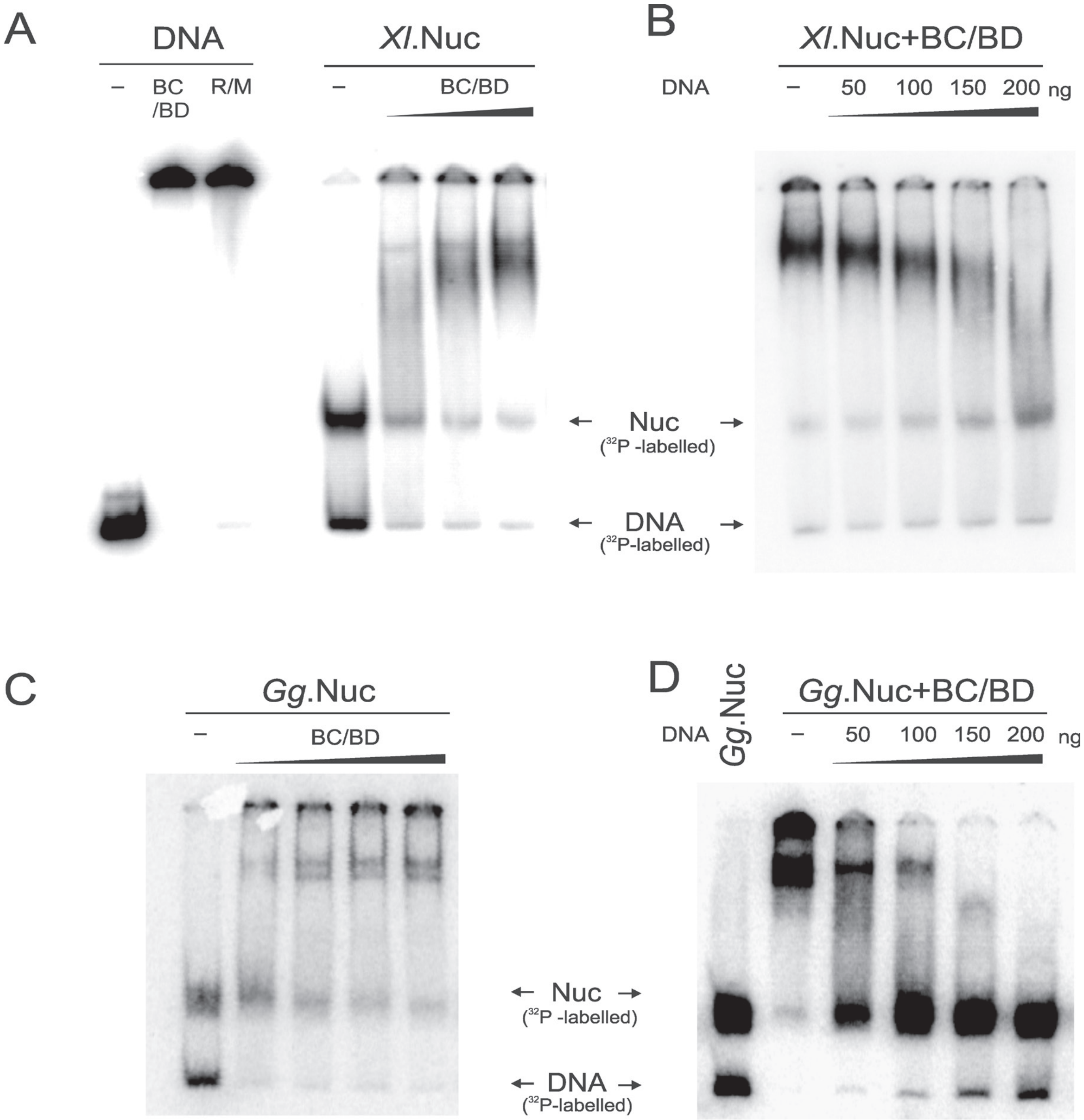


**Figure S1 A-D. BRCA1/BARD1 binds to nucleosomes *in vitro*; Related to Figure 1.** Recombinant full length BRCA1/BARD1 (BC/BD) and RING1B/MEL18 (R/M) were incubated with DNA, recombinant *Xenopus* nucleosomes (**A** and **B**) or nucleosomes reconstituted with chicken erythrocyte octamers (**C** and **D**) and analyzed in an Electrophoretic Mobility Shift Assay (EMSA). For nucleosome reconstitution radiolabelled “601” DNA (161 nt) (Lowary and Widom, 1998) was used. Per reaction approx. 10-40 fmol of gel eluted nucleosomes were incubated with 0.5, 1.0, 1.5 or 2.0 µg of full-length recombinant BRCA1/BARD1 or RING1B/MEL18 in binding buffer (50 mM Tris-HCl pH 7.4, 2.5 mM $MgCl_2$, 0.5 mM DTT). Reactions were loaded on a native TBE polyacrylamide gel (4.5% polyacrylamide, 0.25x TBE). Gels were dried and radiolabelled products were visualized using a Typhoon phosphoimager (GE Healthcare). Competition assays were performed as in the binding reactions described above with 2.0 µg BRCA1/BARD1, followed by the addition of indicated amounts of unlabelled competitor 601 DNA (**B** and **D**).

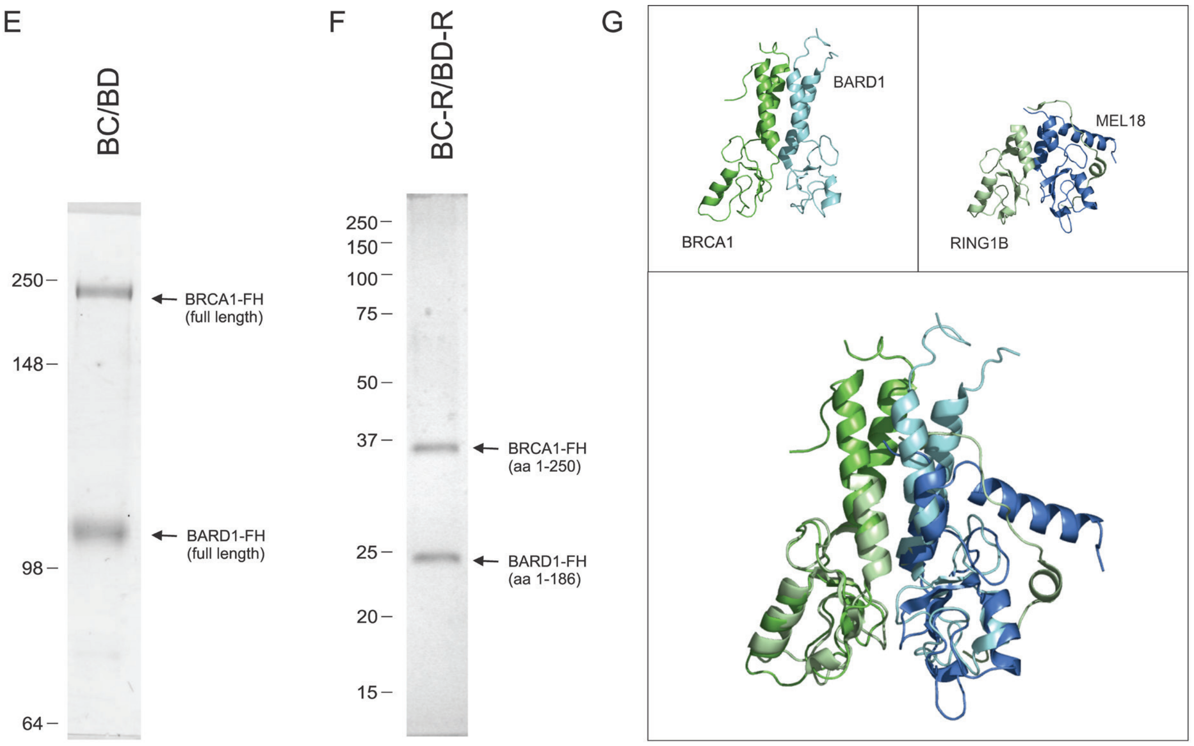


**Figure S1 E-G: BRCA1/BARD1 RING domains share structural and biochemical properties with RING1B/MEL18; Related to Figure 1.** (**E** and **F**) Polyacrylamide gel of recombinant full-length BRCA1/BARD1 (BC/BD) and RING domain complexes comprising residues 1-250 of BRCA1 and 1-186 BARD1 purified from Sf9 cells are shown. (**G**) Structure of the heterodimeric RING complexes formed by BRCA1/BARD1 (top left) and RING1B/MEL18 (top right) based on the diffraction data with the references 2CKL (Buchwald et al., 2006) and 1JM7 (Brzovic et al., 2001). The RING domain structure of MEL18 was predicted using ESyPred3D and visualized in PyMOL (Schroedinger) based on the high degree of identity with its orthologue Bmi1. The structures of BRCA1/BARD1 RING domains superimposed on that of RING1B/MEL18 (bottom) are depicted.

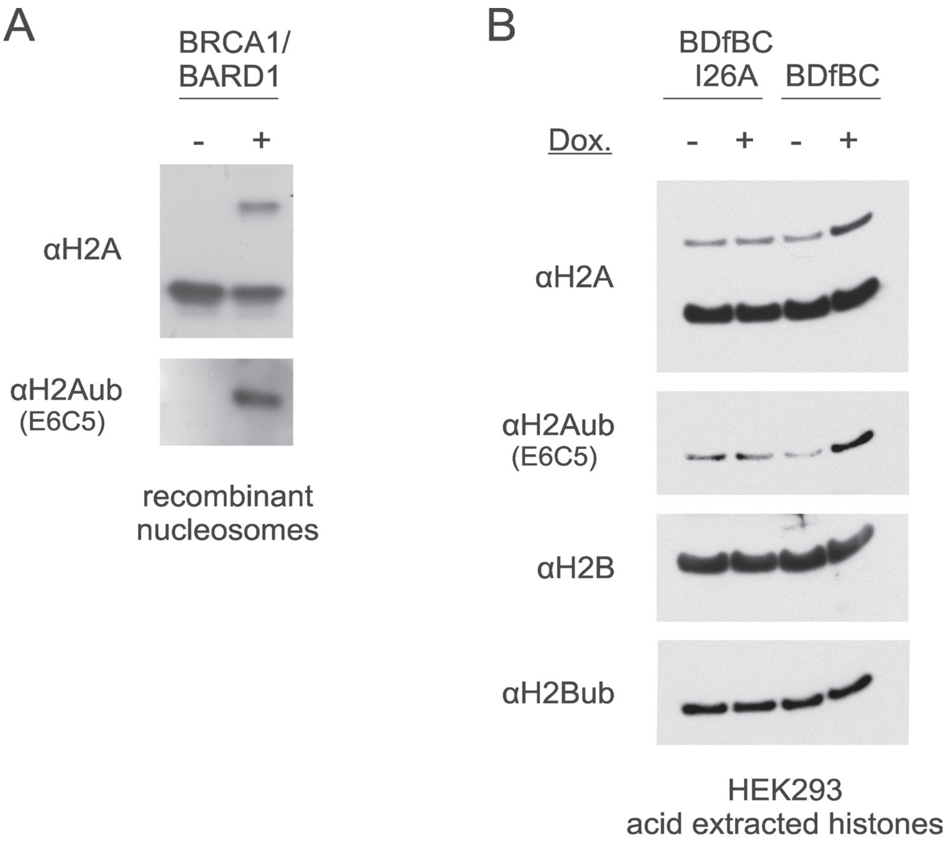


**Figure S2: BRCA1/BARD1 ubiquitylates H2A *in vitro* and *in vivo*; Related to Figure 2.** (**A**) Full length BRCA1/BARD1 ubiquitylates H2A in recombinant nucleosomes *in vitro*. The appearance of a 24 kDa band in an immunoblot using an specific anti-H2A antibody indicates ubiquitylated H2A. Notably, the anti-H2Aub antibody E6C5 detects H2A ubiquitylated by BRCA1/BARD1 in immunblots. (**B**) BDfBC (wt) and BDfBC I26A (mutant) were expressed in stable inducible HEK293 cell lines. 16-24 h after induction of expression with Dox (5µg/ml) cells were harvested. Acid extracted histones were resolved by SDS polyacrylamide gel electrophoresis, transferred to a nitrocellulose membrane and probed with the indicated antibodies.

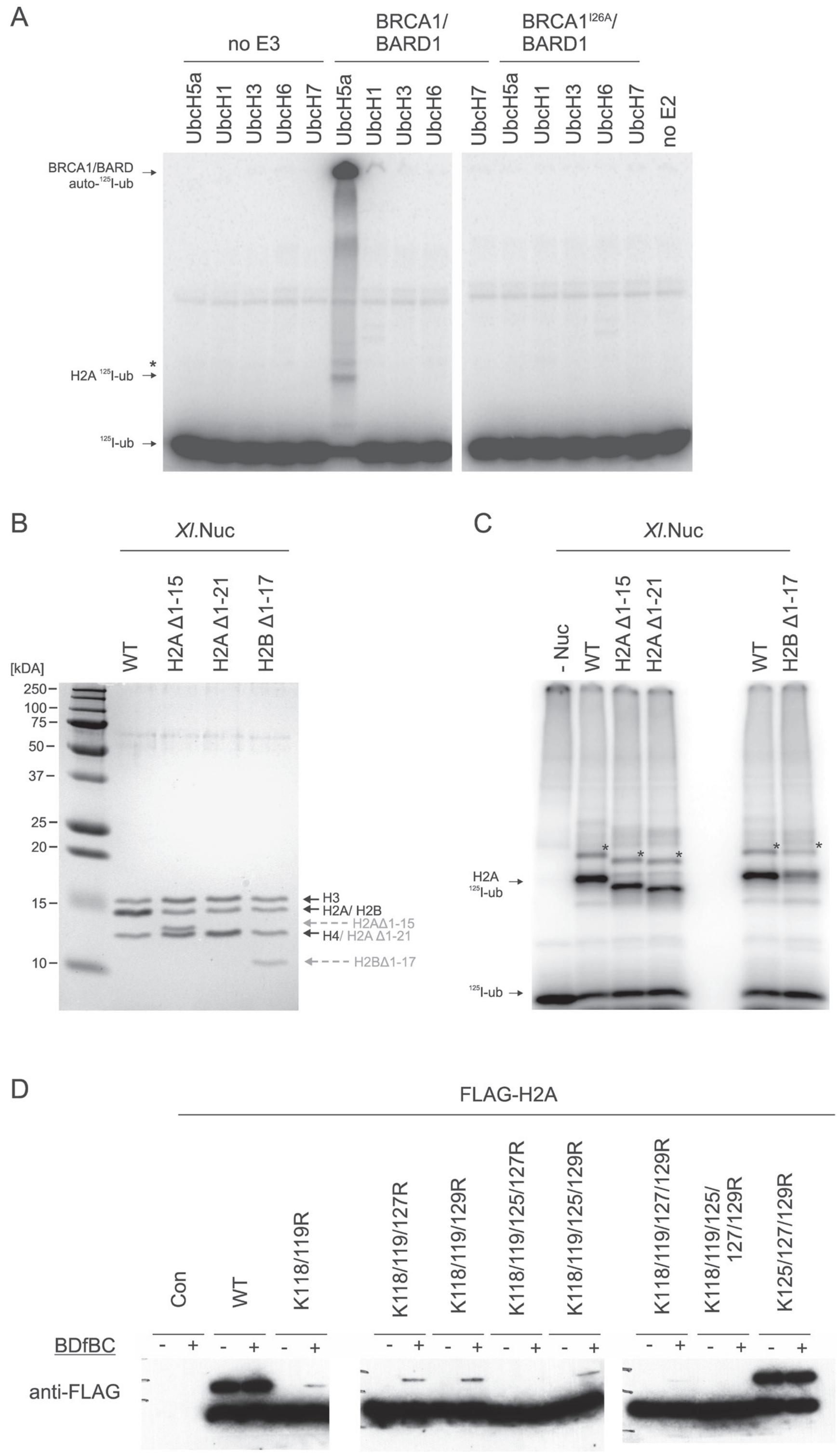


**Figure S3 A-D: BRCA1/BARD1 ubiquitylates H2A at lysines K127-K129, Related to Figure 3.** (**A**) BRCA1/BARD1 ubiquitylates H2A specifically with UbcH5 as the E2 enzyme as indicacted by the appearance of band representing H2A linked with $^{125}$I-labeled ubiquitin. A point mutation in BRCA1 (I26A) which disrupts the binding site for UbcH5 is deficient for H2A ubiquitylation. (**B**) Coomassie stained SDS-PAGE of recombinant nucleosomes containing histones H2A and H2B with small N-terminal deletions as indicated (H2A Δaa1-15 and Δaa1-21, H2B Δaa1-17). (**C**) *In vitro* ubiquitinylation of recombinant nucleosomes with $^{125}$I-labeled ubiquitin. Nucleosomes with deletions in the N-terminal tail of H2A or H2B are indicated. (**D**) HEK293 FlpIn T-Rex cell expressing Tet-inducible BDfBC, were transfected with plasmid expressing FLAG-tagged histone

H2A mutants as indicated. Chromatin was isolated from cells following induction of BDfBC expression by Doxycycline and histones purified by acid precipitation and separated by PAGE as described in Experimental procedures. Ubiquitinylation status of the mutant Histones was monitored by western blot using an anti-FLAG antibody.

E

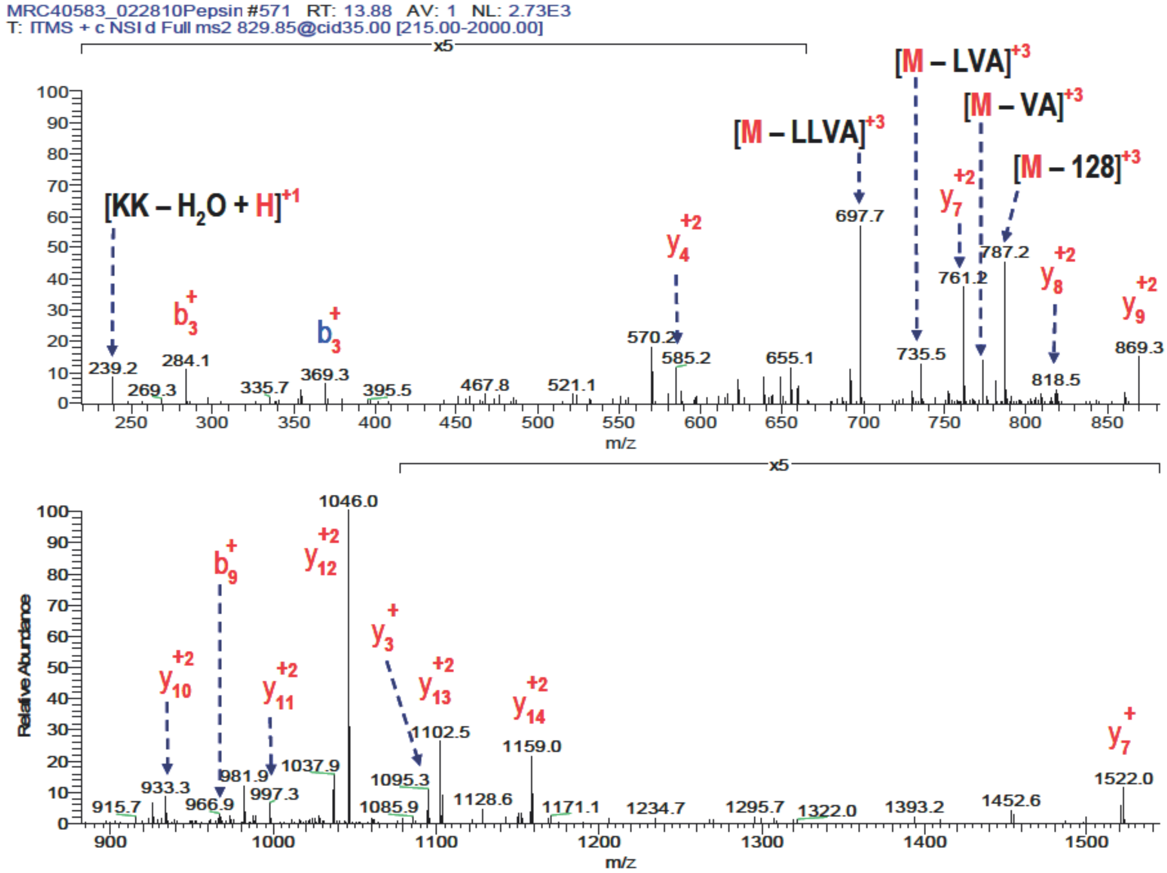

MRC40583_022810Pepsin #571 RT: 13.88 AV: 1 NL: 2.73E3
T: ITMS + c NSI d Full ms2 829.85@cid35.00 [215.00-2000.00]
x5
[KK – H2O + H]+1
[M – LLVA]+3
[M – LVA]+3
[M – VA]+3
[M – 128]+3
b3+
y4+2
y7+2
y8+2
y9+2
b9+
y10+2
y11+2
y12+2
y3+
y13+2
y14+2
y7+
Relative Abundance
m/z


F

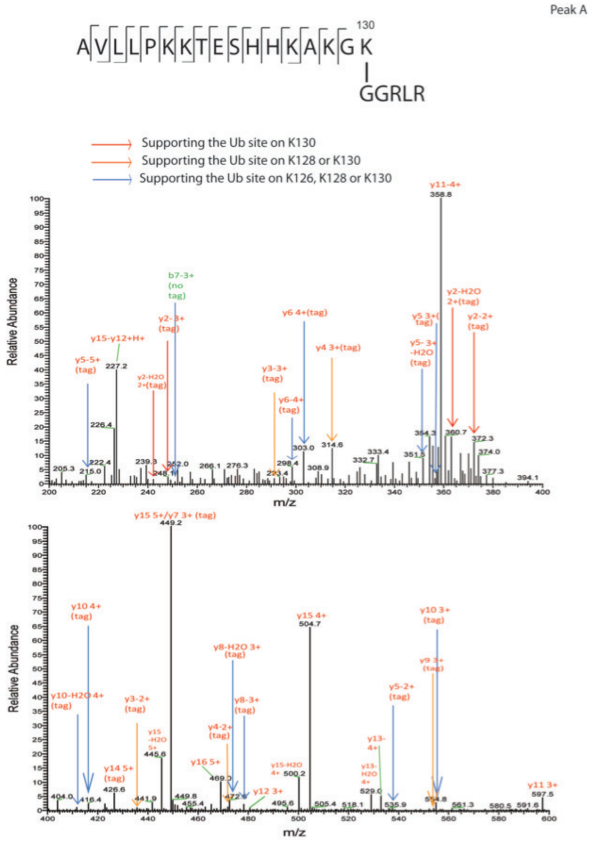

Peak A
A V L L P K K T E S H H K A K G K
130
GGRLR
Supporting the Ub site on K130
Supporting the Ub site on K128 or K130
Supporting the Ub site on K126, K128 or K130
Relative Abundance
m/z


G

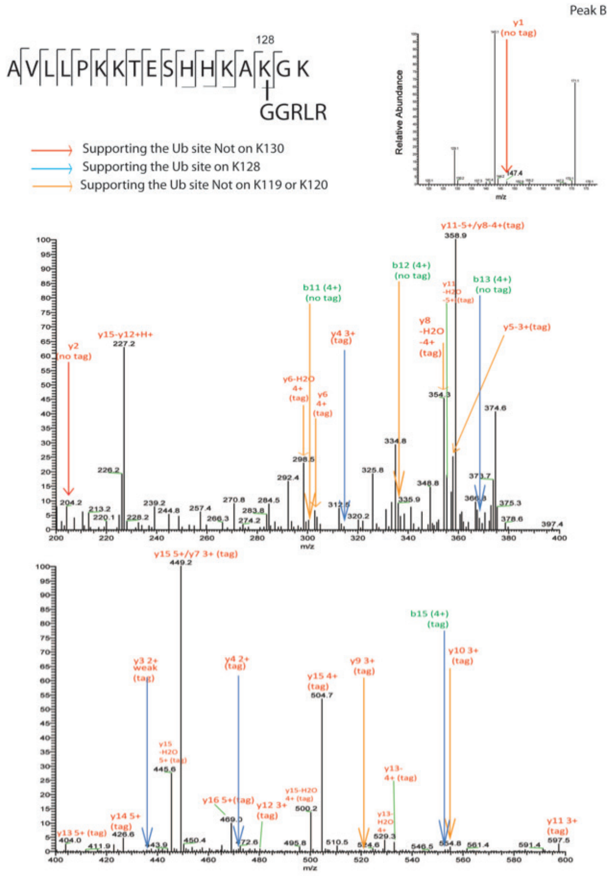

Peak B
A V L L P K K T E S H H K A K G K
128
GGRLR
Supporting the Ub site Not on K130
Supporting the Ub site on K128
Supporting the Ub site Not on K119 or K120
Relative Abundance
m/z

**Fig. S3 E-G: H2A is specifically modified by BRCA1/BARD1 at lysines 127-129, Related to Figure 3G**

(**E**) MS/MS data indicating that K127 of the H2A peptide AVLLPKKTDSHKAKAK is ubiquitinated by BRCA1/BARD1. Chicken chromatin was ubiquitylated by BC-R/BD-R *in vitro*, separated by SDS PAGE. Coomassie-stained H2A and H2Aub bands were excised, digest in-gel with chymotrypsin and pepsin and analysed by mass spectrometry (NextGen Bioscience, Ann Arbor). The peptide depicted was obtained by digestion with pepsin. Comparison of the spectra for unmodified vs ubiquitylated H2A peptide showed that VLRLRGG (ubiquitin) was bound to AVLLPKKTDSHKAKAK (H2A) at lysine 127 (the penultimate lysine) in two independent samples. Five charge states from +3 to +7, m/z = 829.5163 (+3), m/z = 622.3885 (+4), m/z = 498.1120 (+5), m/z = 415.2615 (+6), and m/z = 356.0827 (+7) were observed for this peptide complex. MS/MS data acquired on all these charge states are typical fragmentation patterns for highly protonated peptides and contained both a common set of product ions as well as ions unique to a particular charge state. The complete MS report including y1 is available on request. (**F**, **G**) MS/MS data indicating that K127 and K129 of the H2A peptide AVLLPKKTDSHKAKAK is ubiquitinated by BDfBC *in vivo*. The expression of BDfBC in HEK293 FlpIn T-Rex cell was induced by the addition of Doxycycline for 24 hours. Histones were purified from the cells and separated by PAGE. The region of the gel corresponding to the size of H2Aub was excised, in gel digested with pepsin (pH1.3) and analysed by nanoLC-LTQ/Orbitrap in a data dependent MS/MS mode. Peaks were observed that are consistent with the modification of AVLLPKKTESHHKAKGK peptide with ubiquitin tag (RLRGG). MS/MS spectra for these peaks generated by CID are annotated manually. The MS/MS spectrum shown in e suggests the modification of the K129 residue with RLRGG. A wide range of y ion series depicted by blue and orange arrows indicates that the modification locates at K127 or K129. The four y2 ions (y2(2+), y2(3+), y2-H2O(2+), y2-H2O(3+), red cursors) supports that the modification site is at K129. The MS/MS spectrum in shows a modification of the K127 residue with RLRGG supported by both y and b ion series. The signals matched to b11, b12, b13, b15, y3, y4, y5 and y8 ions suggest that the modification is located at either K127 or K129. In addition, the presence of y1 ion at 147.4, y2 ion at 204.2 and the absence of four y2 ions observed in E suggest that the modification site is not located at K129. K127 is therefore the likely site of modification in **G**. The indicated amino acid positons in E and F refer to the translated cDNA sequence including the post-translationally removed initial methionine.
